# Tuning the Adsorption of H and OH on Ruthenium Aerogel to Boost the Alkaline Hydrogen Evolution

**DOI:** 10.1002/anie.202513970

**Published:** 2025-09-23

**Authors:** Yuanwu Liu, Lirong Wang, Volodymyr Shamraienko, Falk Röder, Angelika Wrzesińska‐Lashkova, Yana Vaynzof, Xiaoming Zhang, Alexander Eychmüller

**Affiliations:** ^1^ Physical Chemistry TU Dresden Zellescher Weg 19 01069 Dresden Germany; ^2^ School of Materials Science and Engineering Hebei University of Technology Tianjin 300130 China; ^3^ Leibniz Institute for Solid State and Materials Research Dresden Helmholtzstraße 20 Dresden 01069 Sachsen Germany; ^4^ Chair for Emerging Electronic Technologies TU Dresden Nöthnitzer Str. 61 Dresden 01187 Sachsen Germany

**Keywords:** Aerogel, Alkaline hydrogen evolution reaction, Doping, Phase regulation, Ruthenium

## Abstract

Ruthenium (Ru) is an ideal alternative to platinum for the alkaline hydrogen evolution reaction (HER) due to its exceptional catalytic performance. However, during the reaction, the excessively strong adsorption of proton (H) and hydroxyl (OH) species on Ru significantly limits its alkaline HER activity. Herein, we enhanced the alkaline HER performance of Ru by modulating its crystalline phase structure and incorporating a low dosage of Cr ions. The synthesized Cr_0.033_Ru_0.967 fcc/hcp_ aerogel exhibits a lower overpotential of only 14 mV at 10 mA cm^−2^, outperforming Ru aerogels (Ru_fcc_ and Ru_hcp_) and commercial Pt/C catalysts. Moreover, Cr_0.033_Ru_0.967 fcc/hcp_ aerogel demonstrates a remarkable stability at 500 mA cm^−2^, sustaining continuous operation for 800 h. Experimental results, combined with theoretical calculations, reveal that the heterogeneous phase interfaces in the CrRu_fcc/hcp_ aerogel effectively weaken the strong adsorption of H at Ru sites. Additionally, with their high affinity for OH species, Cr ions facilitate the removal of OH from adjacent Ru sites. Furthermore, Cr ion incorporation modifies the local electronic structure of Ru, further reducing its OH adsorption energy. This work highlights a synergistic approach to improving catalytic performance and offers a novel strategy for designing highly efficient and durable alkaline HER catalysts.

## Graphical abstract

The formation of Ru hetero‐phases (fcc/hcp) combined with Cr ions doping effectively weakens the strong adsorption of OH and H species on Ru sites. This modulation of adsorption energetics exposes more active Ru centers, thereby significantly enhancing the alkaline HER activity of the Ru aerogel.

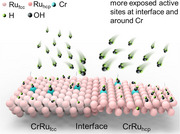

## Introduction

Hydrogen production via water splitting using renewable electricity represents one of the most sustainable and practical solutions to the current energy crisis.^[^ ^1^, ^2^ ^]^ Among the various methods, alkaline water electrolysis remains the predominant industrial process due to its relatively low cost.^[^ ^3^, ^4^ ^]^ Platinum (Pt) is commonly employed as a hydrogen evolution reaction (HER) catalyst, attributed to its superior intrinsic activity. However, the application of Pt in alkaline HER presents several challenges. First, the initial step of the alkaline HER is to adsorb and dissociate water to provide protons for subsequent hydrogen generation.^[^ ^5^, ^6^, ^7^ ^]^ Meanwhile, Pt requires a high energy to decompose water, which results in poor alkaline HER performance.^[^ ^8^, ^9^ ^]^ Second, the high cost of Pt escalates the overall expense of alkaline electrolysis cells.^[^ ^10^ ^]^ Consequently, the development of highly active, Pt‐free catalysts for alkaline HER is paramount for advancing alkaline electrolysis efficiency and affordability.

Ruthenium (Ru) emerges as a promising candidate to replace Pt‐based electrocatalysts for the alkaline HER due to several advantages: 1) stronger water adsorption and lower energy requirements for water decomposition compared to Pt,^[^ ^9^ ^]^ and 2) much lower price than Pt, coupled with strong corrosion resistance in alkaline environments.^[^ ^11^, ^12^, ^13^ ^]^ Nonetheless, the practical application of Ru is hindered by two significant drawbacks. First, Ru exhibits excessively strong H adsorption, impeding H desorption, and thus limiting the HER.^[^ ^14^, ^15^, ^16^, ^17^, ^18^ ^]^ Second, Ru shows strong adsorption of OH species, leading to poisoning of active sites crucial for water adsorption and decomposition.^[^ ^19^ ^]^ To overcome the first limitation, phase engineering has been used to regulate the electronic structure of Ru, which determines the binding strength of reaction intermediates. For instance, Li et al. developed Ru catalysts with heterogeneous phases (hexagonal close‐packed (hcp)/face‐centered cubic (fcc) phase), which altered the electronic structure of interfacial Ru sites to facilitate proton desorption, thereby enhancing the HER performance.^[^ ^20^ ^]^ To mitigate the second limitation, oxophilic species (Cr and SnO_2_) are often combined with Ru, modulating the strong Ru─OH interaction via competitive adsorption between oxophilic species and Ru for OH species.^[^ ^17^, ^21^ ^]^ Notably, Cr exhibits a stronger affinity for OH species due to its *d*‐band center being closer to the Fermi level, making it an ideal promoter for modulating the OH adsorption.^[^ ^22^, ^23^ ^]^ Therefore, doping with Cr atoms and controlling the phase structure of Ru may effectively improve the excessive adsorption of H and OH by Ru, thereby enhancing the HER performance of Ru. However, current synthetic approaches for hetero‐phase Ru remain complex and typically require elevated temperatures. Furthermore, the synergistic combination of heteroatom doping and phase regulation – a promising strategy for simultaneously optimizing H and OH adsorption – still needs to be investigated for Ru‐based HER catalysts.

In this work, we demonstrate the room temperature synthesis of hetero‐phase Ru (Ru_fcc/hcp_) aerogel doped with Cr atoms as a novel electrocatalyst to tune the over‐adsorption of H and OH on Ru aerogel. First, the formation of the heterogeneous phase (fcc/hcp) reduces the H adsorption capacity of Ru sites at the phase interface, enabling rapid H desorption and facilitating hydrogen production. Second, the strong affinity of Cr ions for OH species accelerates the removal of OH species from neighboring Ru sites, reactivating previously deactivated sites. Meanwhile, Cr ions incorporation modifies the local electronic structure of Ru, further reducing its affinity for OH species. Ultimately, these modulation strategies successfully reactivated poisoned Ru sites, significantly enhancing the HER performance and stability of the catalyst. As predicted, the Cr_0.033_Ru_0.967 fcc/hcp_ aerogel exhibited remarkable HER activity, with an extremely low overpotential of 14 and 83 mV at 10 and 300 mA cm^−2^, respectively. Additionally, Cr_0.033_Ru_0.967 fcc/hcp_ aerogel demonstrated excellent stability, maintaining performance for 800 h at a current density of 500 mA cm^−2^.

## Results and Discussion

### Characterization of Ru‐Based Aerogels

**Figure 1:**
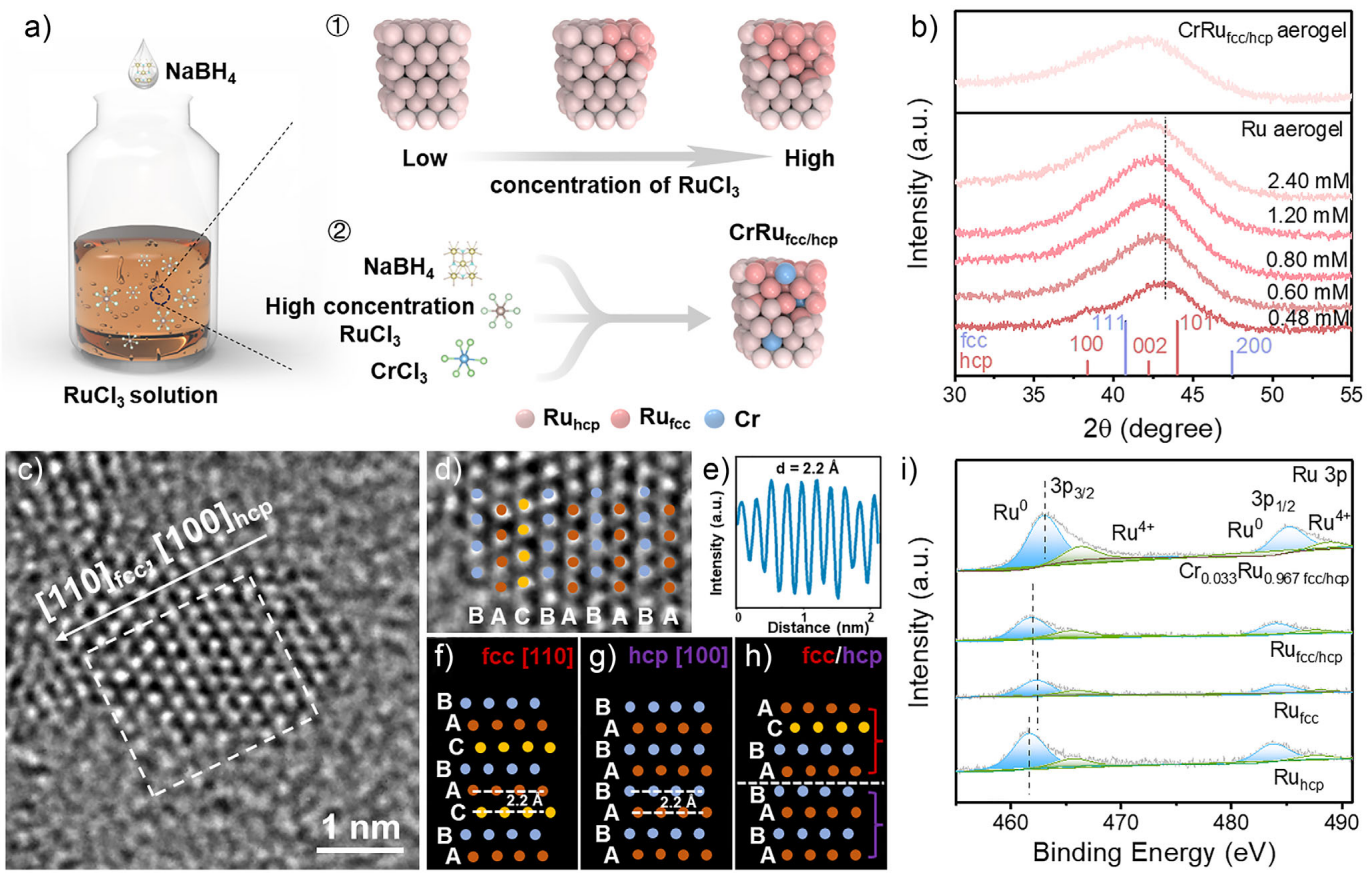
a) Schematic diagram of CrRu_fcc/hcp_ aerogel synthesis. The synthesis process involves two steps: step 1 achieves controlled regulation of Ru crystal structure through modulation of RuCl_3_ precursor concentration; step 2 employs an ion doping strategy to incorporate Cr ions into the lattice structure of Ru aerogel. b) XRD patterns of Ru aerogels and Cr_0.033_Ru_0.967 fcc/hcp_ aerogel. Ru aerogels: Ru aerogels were prepared at different RuCl_3_ precursor concentration (0.48, 0.60, 0.80, 1.2, and 2.4 mM). c) HRTEM images of Ru_fcc/hcp_ aerogel. d) Enlarged image of dashed rectangle in Figure c. The area in the dashed rectangle in c) is rotated about 15 degrees to obtain (d). e) Atomic intensity profile averaged along the short side of the dashed rectangle in (c). Ideal atomic arrangement of Ru viewed from f) hcp [100], g) fcc [110], and h) fcc [110]/ hcp [100]. i) High‐resolution XPS spectra of Ru 3p for Ru‐based aerogels.

The crystalline phase formation of Ru is susceptible to its precursor's reduction kinetics. Rapid reduction conditions favor the formation of the fcc phase, while slower reduction leads to stabilizing the hcp structure.^[^ ^24^ ^]^ Prior studies of Ru aerogels predominantly yielded the hcp phase as the more stable form.^[^ ^11^, ^12^, ^13^ ^]^ This work presents an improved synthetic methodology for preparing mixed‐phase Ru (fcc/hcp) aerogels. As shown in Figure 1a, the formation of Ru_fcc/hcp_ significantly depends on the concentration of RuCl_3_. As the RuCl_3_ concentration increases, the formation time of the Ru aerogel decreases markedly (Figure S1). Specifically, at a RuCl_3_ concentration of 0.48 mM, the formation required 6 h. However, increasing the concentration to 2.4 mM drastically reduced the formation time to just 20 min, indicating a pronounced acceleration in the Ru formation rate with higher precursor concentrations. Low‐magnification transmission electron microscopy (TEM) analysis revealed that despite significant variations in the Ru precursor concentration, the size distribution of Ru nanoparticles constituting the aerogel showed no noticeable alteration (Figure S2). Furthermore, as shown in Figure 1a, adding a Cr ion solution during the synthesis of Ru_fcc/hcp_ aerogels enabled the fabrication of Cr_x_Ru_1‐x fcc/hcp_ aerogels. In Table S1, ICP‐OES results showed that the atomic ratios between Cr and Ru in the series of Cr_x_Ru_1‐x fcc/hcp_ aerogels were 0.008: 0.992, 0.022: 0.978, 0.033: 0.967, and 0.041: 0.959, respectively. Therefore, these aerogels were named Cr_0.008_Ru_0.992 fcc/hcp_, Cr_0.022_Ru_0.978 fcc/hcp_, Cr_0.033_Ru_0.967 fcc/hcp_, and Cr_0.041_Ru_0.959 fcc/hcp_ aerogels. In Figure S3, introducing Cr ions did not significantly affect the morphology and nanoparticle size distribution of Ru_fcc/hcp_ aerogels. Specifically, the average sizes of these nanoparticles are 3.37, 3.70, 3.13, 3.06, and 3.29 nm for Ru_fcc/hcp_, Cr_0.008_Ru_0.992 fcc/hcp_, Cr_0.022_Ru_0.978 fcc/hcp_, Cr_0.033_Ru_0.967 fcc/hcp_, and Cr_0.041_Ru_0.959 fcc/hcp_ aerogels, respectively. The surface area and the pore size distribution of the aerogel were evaluated via Brunauer–Emmett–Teller (BET) physisorption measurements. In Figure S4, the Cr_0.033_Ru_0.967 fcc/hcp_ aerogel exhibits a high specific surface area of 66 m^2^ g^−1^, with pore sizes primarily distributed in the mesoporous range (2–50 nm). The interconnected pore structure will expose more active sites and facilitate mass transport, leading to accelerated reaction kinetics.^[^ ^25^ ^]^

Powder X‐ray diffraction (PXRD) was utilized to investigate the crystal phase of the Ru‐based aerogels. Figure 1b displays the XRD patterns of Ru aerogels synthesized at room temperature with varying RuCl_3_ concentrations of 0.48, 0.60, 0.80, 1.20, and 2.40 mM. At the lowest concentration (0.48 mM), two diffraction peaks were observed at 38.3° and 44.0°, corresponding to the (100) and (101) planes of hcp Ru (JCPDS No. 06‐0663), respectively.^[^ ^26^, ^27^ ^]^ As the RuCl_3_ concentration increased, these peaks gradually merged into a broad peak, with its maximum shifting to 42° in the Ru aerogel synthesized at the highest concentration (2.40 mM). This continuous shift toward the (111) peak of fcc Ru (JCPDS No. 88‐2333) indicates the coexistence of mixed‐phase fcc and hcp Ru. The phase composition of hcp and fcc Ru within the aerogels was further quantified through a PXRD pattern analysis (Figure S5a–f). As the RuCl_3_ concentration increased, the proportion of the unconventional fcc phase progressively rose. However, pure fcc phase Ru aerogel could not be obtained by simply increasing the concentration of RuCl_3_.^[^ ^20^, ^28^ ^]^ To enable a systematic comparison of HER performance among Ru_hcp_, Ru_fcc_, and Ru_fcc/hcp_ in subsequent sections, the Ru_fcc_ aerogel was successfully synthesized through a chemical reduction approach (Figure S6). Additionally, as shown in Figures 1b and S4g–h, Cr ion doping did not significantly alter the XRD peaks and phase ratio of the Ru_fcc/hcp_ aerogel (RuCl_3_ concentration: 2.4 mM), likely due to the low concentration of Cr ions incorporated during the synthesis process.

High‐resolution TEM (HRTEM) image reveals detailed structural insights into the Ru_fcc/hcp_ aerogel. Figure 1c displays the atomic arrangement of a suitably oriented nanoparticle. An enlarged view (Figure 1d) confirms the predominance of the hcp phase of Ru, characterized by its ABAB… stacking sequence of close‐packed planes. Additionally, regions with the fcc phase Ru (ABCABC… stacking sequence) are observed within the nanoparticle. As illustrated in Figure 1e, the measured lattice spacing of 2.2 Å corresponds to the (002) plane of hcp Ru and the (111) plane of fcc Ru, respectively.^[^ ^24^, ^29^ ^]^ For further validation, simulated atomic arrangements of the fcc phase along the [110] direction and the hcp phase along the [100] direction (Figure 1f–h) match the experimental observations. These findings conclusively demonstrate the formation of a mixed‐phase Ru_fcc/hcp_ structure. In Figure S7a–d, Energy‐dispersive X‐ray spectroscopy (EDXs) and scanning transmission electron microscopy‐ electron energy loss spectroscopy (STEM‐EELS) analysis confirm the presence of Ru alongside detectable Cr in the CrRu_fcc/hcp_ aerogel, indicating the Cr ions were successfully introduced into Ru_fcc/hcp_ aerogel. Additionally, in Figure S7d, the peaks at around 532 eV corresponding to O‐K edge were also observed, suggesting a partial oxidation of metallic Ru upon exposure to air.

To investigate the evolution of the Ru crystalline phase and the impact of Cr ions doping on the electronic structure of Ru aerogels, a systematic characterization was performed using zeta potential analysis, ultraviolet photoemission spectroscopy (UPS) and X‐ray photoemission spectroscopy (XPS). The Zeta potential analysis was first employed to examine the surface charge properties of Ru aerogels. As illustrated in Figure S8, the zeta potential values of mixed‐phase Ru aerogels exhibit a notable shift compared to those of hcp and fcc Ru aerogels. Moreover, the incorporation of Cr ions further amplifies this shift. In Figure S9, UPS results indicate a minor decrease in the work functions among the Ru_hcp_ (4.5 eV), Ru_fcc/hcp_ (4.45 eV), and CrRu_fcc/hcp_ (4.4 eV). However, this change falls within the experimental error of the measurement. XPS was further employed to analyze the changes in electronic states of Ru aerogels. As shown in Figure 1i, high‐resolution XPS spectra of the Ru 3p region revealed spin‐orbit‐split doublets, with peaks at 461.7 and 483.8 eV corresponding to Ru 3p_3/2_ and Ru 3p_1/2_ of metallic Ru, respectively.^[^ ^30^, ^31^, ^32^, ^33^, ^34^ ^]^ Weaker peaks with binding energies of 465.8 and 487.8 eV corresponding to the oxidized Ru species were also observed, suggesting a partial oxidation of metallic Ru upon exposure to air.^[^ ^15^, ^35^ ^]^ Interestingly, the binding energy of Ru 3p in Ru_fcc/hcp_ was located between Ru_hcp_ and Ru_fcc_, confirming the electronic structure was optimized by the phase regulation. For CrRu_fcc/hcp_ aerogel, the binding energy of Ru 3p positively shifted compared with Ru_fcc/hcp_ aerogel, indicating doping Cr ions in Ru_fcc/hcp_ aerogels can modulate the electronic structure of Ru. This optimized electronic structure of CrRu_fcc/hcp_ aerogel may optimize its adsorption strength for HER reaction intermediates, thus improving the HER performance of Ru. In addition, the Cr 2p spectra exhibited a weak peak at 576.3 eV, indicating the presence of Cr in all Cr_x_Ru_1‐x fcc/hcp_ aerogels (Figure S10).^[^ ^36^ ^]^

### HER Activity

**Figure 2:**
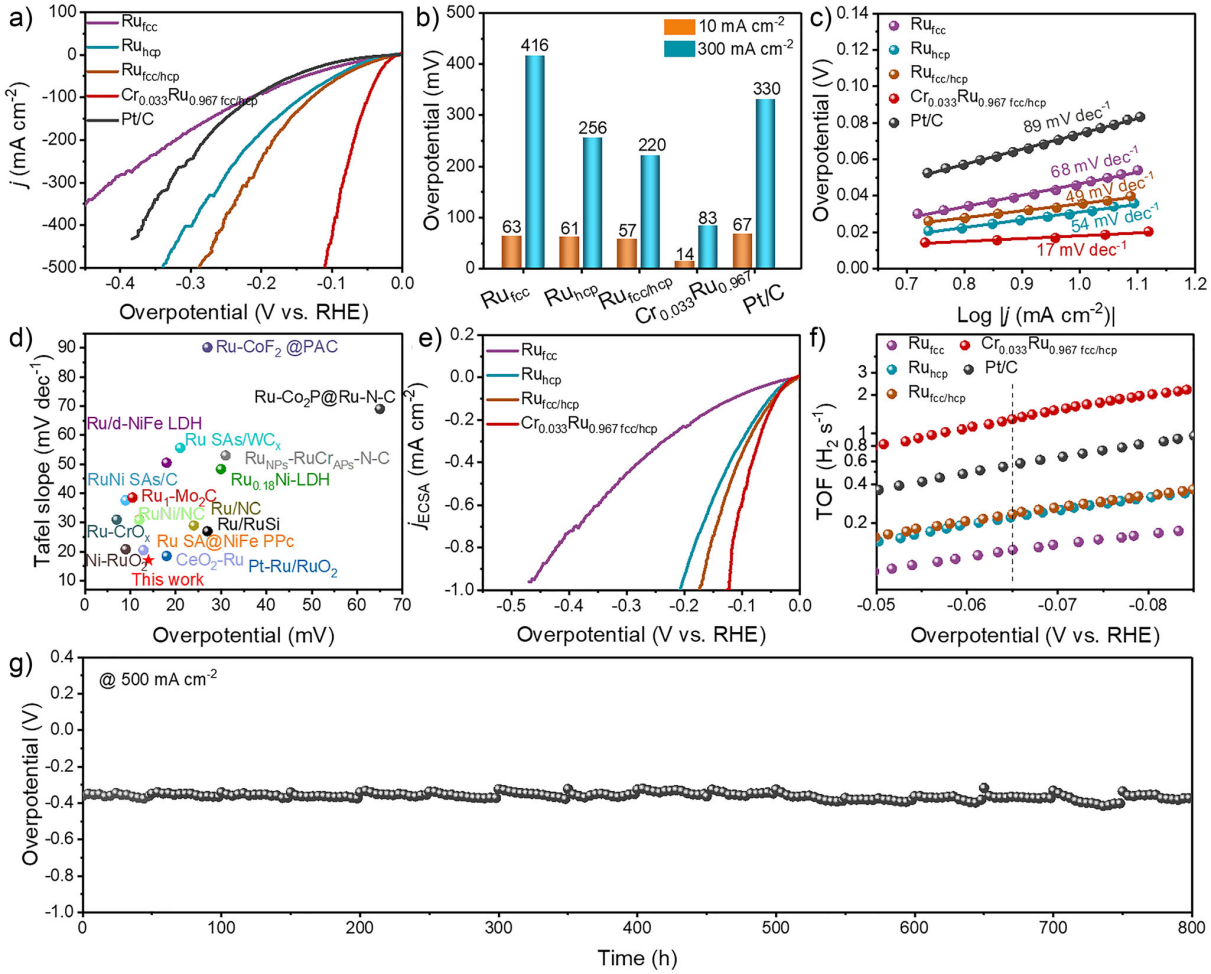
a) Polarization curves of Ru‐based aerogels and Pt/C with 90% iR correction. b) Comparison of overpotentials of Ru‐based aerogels and Pt/C at 10 and 300 mA cm^−2^. c) Corresponding Tafel slopes. d) Comparisons in overpotentials and Tafel slopes of Cr_0.033_Ru_0.967 fcc/hcp_ aerogel and other reported Ru‐based catalysts. e) Polarization curves of Ru‐based aerogels normalized by ECSA. f) TOF values of Ru‐based aerogels and Pt/C. g) Stability measurement for Cr_0.033_Ru_0.967 fcc/hcp_ aerogel under a current density of ‐500 mA cm^−2^ in 1 M KOH.

The HER activity of the Ru‐based aerogels was first evaluated by using a standard three‐electrode system in 1.0 M KOH electrolyte. To fully demonstrate the unique structural advantages of aerogels, this study systematically compared the electrochemical performance of Ru_fcc_ aerogel with Ru_fcc_ nanoparticles. In Figure S11a–e, the results revealed that the Ru_fcc_ aerogel exhibits a significantly larger electrochemical active surface area (ECSA) than its nanoparticle counterpart. Benefiting from this improved active area, in Figure S11f, the Ru_fcc_ aerogel demonstrates good HER performance in alkaline media, exhibiting a lower overpotential of 63 mV at 10 mA cm^−2^ compared to nanoparticles (185 mV). These results conclusively validate the unique advantages of the three‐dimensional porous aerogel structure in exposing active sites and facilitating mass transport. Subsequently, the alkaline HER performance of Ru aerogels with different crystalline phases and Cr_x_Ru_1‐x_ series aerogels was evaluated. As demonstrated by polarization curves in Figures 2a,b and S12a,b, compared to Ru_hcp_ and Ru_fcc_, the mixed‐phase Ru_fcc/hcp_ exhibits lower overpotential at a current density of 10 mA cm^−2^, demonstrating that tailoring the crystal phase structure of Ru can enhance its HER performance. Furthermore, doping mixed‐phase Ru_fcc/hcp_ with Cr ions further decreases the overpotential at the same current density. Among these samples, the Cr_0.033_Ru_0.967 fcc/hcp_ aerogel exhibits an ultralow overpotential of 14 mV at current densities of 10 mA cm^−2^, which is much lower than that of Cr_0.008_Ru_0.992 fcc/hcp_ (21 mV), Cr_0.022_Ru_0.978 fcc/hcp_ (17 mV), Cr_0.041_Ru_0.959 fcc/hcp_ (56 mV), Ru_fcc/hcp_ (57 mV), and commercial Pt/C (67 mV). Impressively, at a higher current density of 300 mA cm^−2^, the Cr_0.033_Ru_0.967 fcc/hcp_ aerogel still possesses the lowest overpotential (83 mV) among all studied electrocatalysts, indicating a significant role of the Cr sites for accelerating the alkaline HER processes. Moreover, Figures 2c and S12c illustrate the Tafel slope based on the polarization curves and the corresponding values are 22, 17, 17, 42, 68, 54, 49, and 89 mV dec^−1^ for Cr_0.008_Ru_0.992 fcc/hcp_, Cr_0.022_Ru_0.978 fcc/hcp_, Cr_0.033_Ru_0.967 fcc/hcp_, Cr_0.041_Ru_0.959 fcc/hcp_, Ru_fcc_, Ru_hcp_, Ru_fcc/hcp_ aerogels, and commercial Pt/C, respectively. The lowest Tafel slope of the Cr_0.033_Ru_0.967 fcc/hcp_ aerogel indicates the most effective facilitation of the water dissociation and hydrogen evolution kinetic. More importantly, the low Tafel slope of the above Ru‐based materials indicates that the rate‐determining step of the reaction is the desorption of the OH intermediate, rather than the water dissociation step. In order to further highlight the advantages of overpotential and Tafel slope, a synopsis of performances on recently reported Ru‐based HER electrocatalysts in alkaline media is listed in Figures 2d and Table S2, in which the Cr_0.033_Ru_0.967 fcc/hcp_ aerogel is superior to some others. The excellent performance of Cr_0.033_Ru_0.967 fcc/hcp_ aerogel was further confirmed through four independent experiments (Figure S13).

The mass activity, a critical metric for assessing the catalyst utilization efficiency, holds particular significance in evaluating noble metal‐based catalysts. As illustrated in Figure S14, a comparative analysis of mass‐normalized activities was conducted for both Ru‐based aerogels and Pt/C catalysts. Notably, the Cr_0.033_Ru_0.967 fcc/hcp_ aerogel demonstrates remarkable catalytic performance, achieving a mass activity of 1.79 A mg^−1^, with its performance reaching five times that of the commercial Pt/C catalyst (0.36 A mg^−1^). The ECSA of Ru‐based aerogels and Pt/C was determined to quantify variations in their active site densities (Figures S15 and S16a–c). The results reveal that constructing hetero‐phase structures in Ru aerogels and incorporating an appropriate amount of Cr ions effectively enhance the number of surface catalytically active sites. To gain deeper insights into the intrinsic activity of the Ru‐based catalysts and Pt/C, polarization curves normalized by ECSA and turnover frequency (TOF) were obtained (Figures 2e,f and S16d). The ECSA‐normalized data demonstrate that the Cr_0.033_Ru_0.967 fcc/hcp_ aerogel maintains excellent current density compared to other Ru‐based catalysts and Pt/C, while its TOF value (1.29 H_2_ s^−1^ at ‐0.065 V) is also the highest. These findings confirm that the synergistic effects of heterogeneous phase formation and Cr ion incorporation substantially boost the number of active sites and the inherent catalytic activity of Ru‐based systems. Additionally, electrochemical impedance spectroscopy (EIS) obtained from the corresponding equivalent circuit diagram was utilized to explore the charge transfer capacity, in which the Cr_0.033_Ru_0.967 fcc/hcp_ aerogel showed the lowest resistance, indicating an optimal conductivity of the Cr_0.033_Ru_0.967 fcc/hcp_ aerogel toward the HER (Figure S17). To validate the electron utilization efficiency during the HER, we calculated the Faradaic efficiency. As shown in Figure S18, the Faradaic efficiency of the HER is close to 100%, indicating excellent electrochemical selectivity and the absence of significant side reactions. Apart from the activity, durability is also critical to assess HER electrocatalysts. As shown in Figure 2g, the Cr_0.033_Ru_0.967 fcc/hcp_ aerogel exhibits excellent stability at a current density of 500 mA cm^−2^ for 800 h. Post‐reaction TEM imaging confirms that the overall morphology of Cr_0.033_Ru_0.967 fcc/hcp_ aerogel was well preserved (Figure S19a). Additionally, after prolonged high current density stability testing, the XRD pattern revealed a decrease in the proportion of fcc Ru, indicating a partial phase transformation from fcc to hcp. This phase transition is likely the reason for the little decline in stability after 800 h. (Figure S19c,d).

### Improved HER Mechanism

**Figure 3:**
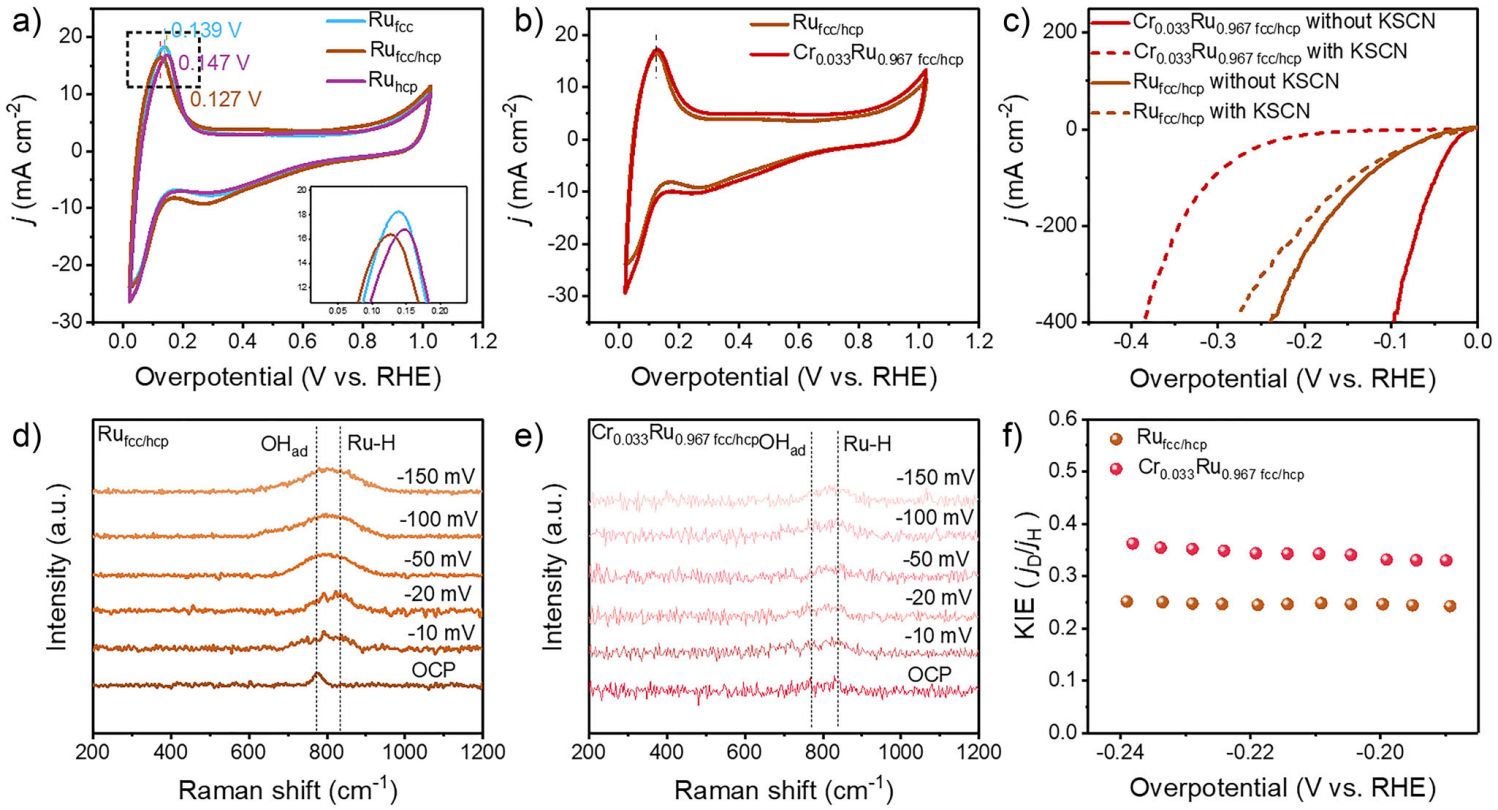
CV curves of a) Ru_fcc_, Ru_hcp_, and Ru_fcc/hcp_ aerogels, and b) Ru_fcc/hcp_ and Cr_0.033_Ru_0.967 fcc/hcp_ aerogels. c) Polarization curves of Ru_fcc/hcp_ and Cr_0.033_Ru_0.967 fcc/hcp_ aerogels measured in 1 M KOH with or without KSCN. In situ Raman spectra of d) Ru_fcc/hcp_ and e) Cr_0.033_Ru_0.967 fcc/hcp_ aerogels at different potentials. f) KIE values at different potentials of Ru_fcc/hcp_ and Cr_0.033_Ru_0.967 fcc/hcp_ aerogels.

The hydrogen binding energy (HBE) of the catalyst was investigated by desorption of the underpotential deposited hydrogen (H_upd_). In Figure 3a, compared to Ru_hcp_ and Ru_fcc_, the H_upd_ peak potential of Ru_fcc/hcp_ shifts significantly toward lower potentials, indicating a weaker binding between H and the active sites on the Ru_fcc/hcp_ surface, which is more conducive to the desorption of H. To further validate the promoting effect of crystal phase regulation on H desorption and hydrogen generation, we applied Ru_hcp_, Ru_fcc_, and Ru_fcc/hcp_ to acidic HER testing. Since the acidic HER is solely related to the adsorption and desorption processes of H, this test can effectively reflect the influence of the crystal phase on the HER performance.^[^ ^37^ ^]^ In Figure S20, the activity of Ru_fcc/hcp_ in the HER is significantly better than that of Ru_hcp_ and Ru_fcc_, confirming that the desorption of H on the Ru surface indeed promotes the HER. However, it is noteworthy that in Figure 3b, the H_upd_ peak potential of Cr_0.033_Ru_0.967 fcc/hcp_ does not show a significant shift compared to Ru_fcc/hcp_, indicating that the introduction of low‐dose Cr ions does not effectively weaken the adsorption of H on Ru. When the concentration of Cr increases to 4.1% in the CrRu_fcc/hcp_ aerogel, the H_upd_ peak potential shifts toward higher potential compared to Ru_fcc/hcp_ (Figure S21), indicating that the introduction of large number of Cr ions enhances the interaction strength between H and the active sites on the Ru_fcc/hcp_ surface.

To further investigate the role of Cr ions in enhancing the HER performance, we designed poisoning experiments to identify active centers. Thiocyanate ions (SCN^−^), toxic to metal‐centered active sites, were introduced into the electrolyte.^[^ ^38^ ^]^ In Figure 3c, the Ru_fcc/hcp_ aerogel shows negligible current decay, which suggests that SCN^−^ ions have almost no poisoning effect on the Ru_fcc/hcp_ aerogel. This phenomenon can be attributed to the surface‐adsorbed OH groups, which effectively counteract the poisoning of SCN^−^. Previous studies have also confirmed that Ru's exceptionally strong OH^−^ adsorption can block its surface sites, making it difficult for other species to adsorb.^[^ ^27^, ^39^ ^]^ However, the Cr_0.033_Ru_0.967 fcc/hcp_ aerogel shows an obvious current decay, demonstrating that Cr ions can help to desorb OH from the Ru aerogel surface, thereby exposing Ru sites. In situ Raman spectroscopy was further used to check the OH adsorption ability on Ru_fcc/hcp_ and Cr_0.033_Ru_0.967 fcc/hcp_ aerogels. Figure 3d shows a peak for the Ru_fcc/hcp_ aerogel at 774 cm^−1^ under open circuit potential (OCP) conditions. Deuterium isotope experiments were performed to identify the species observed in Figure 3d. As shown in Figure S22, the peak at 774 cm^−1^ is shifted to 702 cm^−1^, which means that the intermediate product contains H atoms and can be attributed to OH_ad_ species.^[^ ^40^ ^]^ In Figures 3d and S23, the OH_ad_ peak intensity of Ru_fcc/hcp_ aerogel was significantly enhanced with the negative shift of the potential, which indicated that the OH_ad_ generated by water cleavage failed to desorb efficiently and continued to occupy the active site of Ru. Meanwhile, the presence of Ru–H species was also detected and its peak intensity increased with the negative potential shift, indicating the strong adsorption of H on Ru sites. In contrast, the intensity of the OH_ad_ peak of the Cr_0.033_Ru_0.967 fcc/hcp_ aerogel remained stable with the gradual increase of the potential in the negative direction, indicating that the OH_ad_ generated by the decomposition of water molecules on its surface could be desorbed in time (Figures 3e and S24). This comparison further reveals the enhancement of the OH_ad_ desorption ability on the surface of Cr_0.033_Ru_0.967 fcc/hcp_ aerogel and its facilitation of the HER process. The CO stripping is another method to investigate catalysts' OH adsorption capability. In Figure S25, the CO oxidation peak potential shows a positive shift for the Cr_0.033_Ru_0.967 fcc/hcp_ aerogel compared to Ru_fcc/hcp_ aerogel, indicating that the adsorption of OH on the Cr_0.033_Ru_0.967 fcc/hcp_ aerogel surface is weakened. Kinetic isotope effect (KIE) measurements in 1.0 M KOD/D_2_O and KOH/H_2_O revealed Cr_0.033_Ru_0.967 fcc/hcp_’s higher KIE value versus Ru_fcc/hcp_, indicating enhanced water dissociation and faster Volmer step kinetics in the HER.

**Figure 4:**
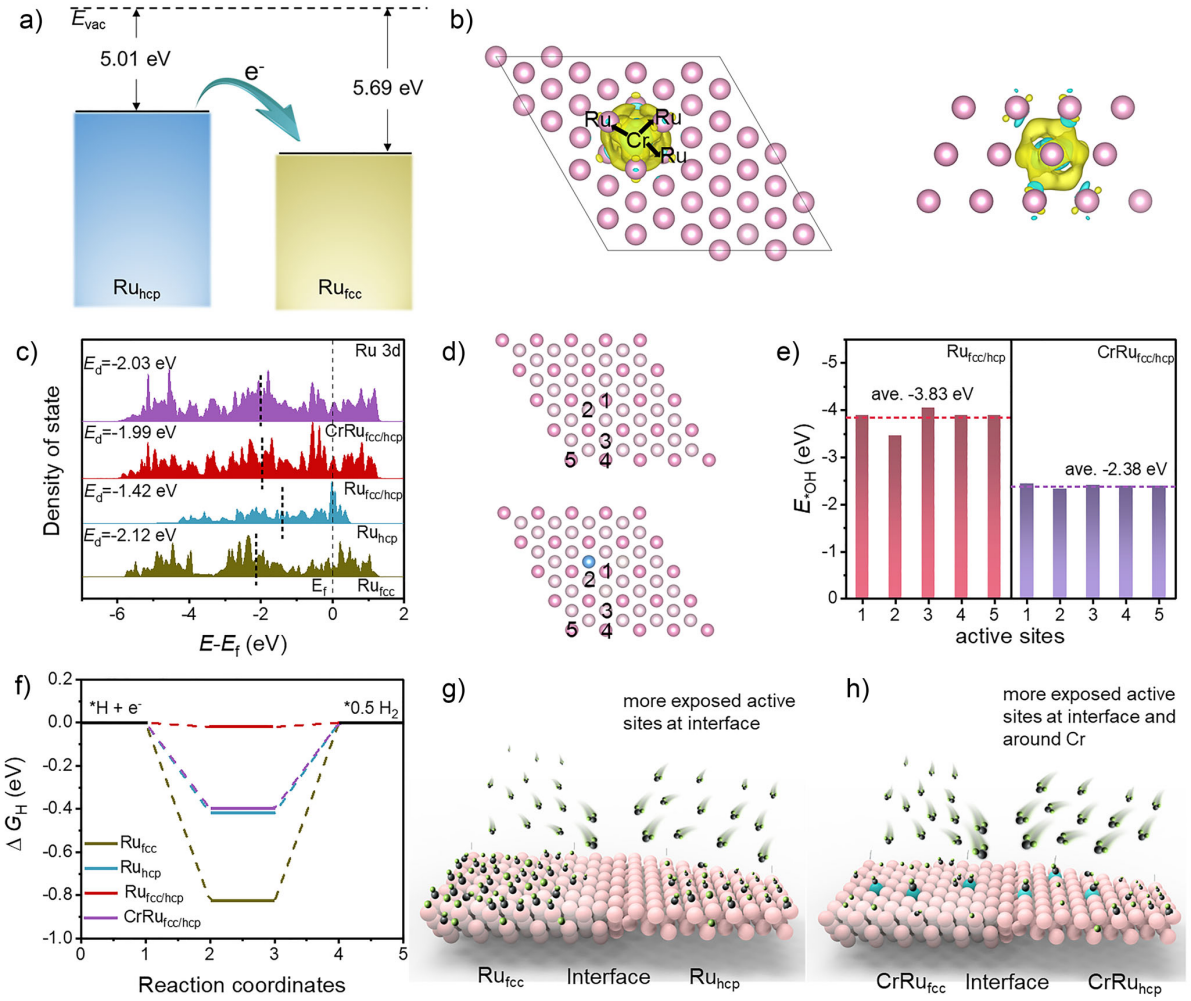
a) Work function diagram of Ru_hcp_ and Ru_fcc_. b) Differential charge density image of CrRu_fcc/hcp_. Yellow and blue regions reflect charge accumulation and depletion, respectively. c) Density of states of the *d* orbitals for Ru_hcp_, Ru_fcc_, Ru_fcc/hcp_, and CrRu_fcc/hcp_. The dashed line at 0 represents the Fermi level; The short black dashed lines represent the location of the *d*‐band center. d), e) OH adsorption strength of different sites at Ru_fcc/hcp_ and CrRu_fcc/hcp_. f) Gibbs free energy of H adsorption for Ru_hcp_, Ru_fcc_, Ru_fcc/hcp_, and CrRu_fcc/hcp_. g), h) Schematic diagram of improved HER mechanism for Ru_fcc/hcp_ and CrRu_fcc/hcp_. Pink, blue, black, and green balls represent Ru, Cr, O, and H, respectively.

To understand the effect of the crystal phase modulation and Cr ion doping on the alkaline HER performance of Ru from the energy level, Density Functional Theory (DFT) calculations were performed. First, the structure models of Ru_fcc_, Ru_hcp_, Ru_fcc/hcp_, and CrRu_fcc/hcp_ were constructed (Figures S26 and S27). In Figure 4a, the work function of Ru_hcp_ and Ru_fcc_ are 5.01 and 5.69 eV, respectively, leading to the redistribution of electrons at the fcc and hcp Ru interface. When Cr is doped into Ru, the electronic structure of the Cr atom and the surrounding Ru atoms is redistributed, the electron density of the Cr atoms decreases, and the electron density of the surrounding Ru atoms increases (Figure 4b). The change in the electronic structure of Ru atoms may cause the shift of their *d*‐band center, thereby changing the adsorption strength of Ru atoms and reaction intermediates. Therefore, the projected density of states of Ru_hcp_, Ru_fcc_, Ru_fcc/hcp_, and CrRu_fcc/hcp_ were analyzed (Figure 4c). Based on Figure 4c, the location of the *d*‐band center of the catalysts was calculated. The *d*‐band center of Ru_fcc_ is positioned farthest from the Fermi level, indicating its weakest adsorption capability toward reaction intermediates.^[^ ^19^ ^]^ In contrast, the *d*‐band center of Ru_hcp_ is closest to the Fermi level, suggesting its strongest adsorption capability toward reaction intermediates. The *d*‐band center of Ru_fcc/hcp_ lies between those of Ru_fcc_ and Ru_hcp_, demonstrating a moderate adsorption strength toward reaction intermediates, which is neither too strong nor too weak. Notably, the *d*‐band center of CrRu_fcc/hcp_ shifts slightly further away from the Fermi level compared to that of Ru_fcc/hcp_, implying a further reduction in its adsorption capability toward reaction intermediates. In order to investigate the mechanism of the effect of Cr introduction on the adsorption behavior of Ru with OH_ad_, we systematically analyzed the adsorption energy of OH_ad_ on the surfaces of Ru_fcc/hcp_ and CrRu_fcc/hcp_ (Figure 4d,e). In Figure 4e, the average adsorption energy of OH_ad_ on CrRu_fcc/hcp_ was significantly reduced compared with that on Ru_fcc/hcp_. This result is consistent with the in situ Raman analysis (Figures 3, S23, and S24). Further analysis revealed that the adsorption energy of the Cr sites for OH_ad_ was larger than most of Ru atoms in the system (Figure S28), indicating a competitive OH adsorption between the Cr sites and the surrounding Ru atoms. In addition, we systematically analyzed the H adsorption Gibbs free energy (Δ*G*_H*_) of different Ru‐based aerogels. As shown in Figure 4f, the Δ*G*_H*_ value of Ru_fcc/hcp_ is the closest to the ideal value of zero, indicating that the formation of heterogeneous phase structure can significantly weaken the desorption ability of Ru to H. This finding agrees well with the aforementioned results of the *d*‐band center analysis, acidic HER performance tests and HBE analysis (Figures 3, 4, and S20). However, the introduction of Cr causes the Δ*G*_H*_ value of Ru to be far away from the zero again, suggesting that Cr doping enhances the adsorption of Ru on H. This is consistent with the HBE analysis results (Figures 3b and S21), which show that the introduction of a large number of Cr atoms leads to enhanced adsorption of H by Ru.

The Ru_hcp_ aerogel is significantly limited in its alkaline HER performance due to the strong adsorption of OH_ad_ and H_ad_, which results in many occupied active sites on its surface. In Figure 4g, the formation of the heterogeneous phase Ru (Ru_fcc/hcp_) optimized the electronic structure of Ru atoms at the phase interface, effectively weakened its adsorption strength for OH_ad_ and H_ad_, and allowed a large number of active sites in the interface region to be fully exposed. Through competitive adsorption effects, the introduction of Cr ions significantly reduces the adsorption energy of OH_ad_ species on Ru active sites in non‐interfacial phases.

## Conclusions

In summary, we successfully synthesized a CrRu_fcc/hcp_ aerogel with exceptional performance through a synergistic strategy of crystal phase regulation and atomic doping. Experimental results demonstrate that this strategy effectively modulates the adsorption behavior of H_ad_ and OH_ad_ on the Ru surface. Specifically, the formation of a Ru heterophase (fcc/hcp) optimizes the electronic structure of Ru at the phase interface, significantly reducing its adsorption strength for H and bringing its Δ*G*_H*_ close to zero. Meanwhile, the introduction of Cr ions modulates the local electronic structure of Ru, facilitating the desorption process of OH_ad_ from Ru sites, thereby effectively reactivating previously poisoned active sites. Benefiting from these synergistic effects, the CrRu_fcc/hcp_ aerogel exhibits significantly enhanced HER activity under alkaline conditions and excellent long‐term stability at high current densities.

## Supporting Information

The supporting information is available free of charge.

Experimental and computation methods, chemicals, material characterizations, electrochemical tests.

## Conflict of Interests

The authors declare no conflict of interest.

## Supporting information



Supporting Information

## Data Availability

The data that support the findings of this study are available from the corresponding author upon reasonable request.
