# Supplementary material for: Tuning the Adsorption of H and OH on Ruthenium Aerogel to Boost the Alkaline Hydrogen Evolution
Source: Angew Chem Int Ed Engl. 2025 Sep 23;64(47):e202513970. doi: 10.1002/anie.202513970 (PMC12624330; doi:10.1002/anie.202513970)
Supplement: Supplementary file 1 — Supporting Information [file ANIE-64-e202513970-s001.docx]

**Tuning the Adsorption of H and OH on Ruthenium Aerogel to Boost the Alkaline Hydrogen Evolution**

*Yuanwu Liu^a╂^, Lirong Wang^b╂^, Volodymyr Shamraienko^a^,* *Falk Röder^c^, Angelika Wrzesińska-Lashkova^c,d^, Yana Vaynzof^c,d^, Xiaoming Zhang^b^*, and Alexander Eychmüller^a^**

^a^ Physical Chemistry, TU Dresden, Zellescher Weg 19, 01069 Dresden, Germany

*E-mail: [alexander.eychmueller@tu-dresden.de](mailto:alexander.eychmueller@tu-dresden.de)

^b^ School of Materials Science and Engineering, Hebei University of Technology, Tianjin 300130, China

*E-mail: [zhangxiaoming87@hebut.edu.cn](mailto:zhangxiaoming87@hebut.edu.cn)

^c^ Leibniz Institute for Solid State and Materials Research, Dresden, Helmholtzstraße 20, Dresden, 01069 Sachsen, Germany

^d^ Chair for Emerging Electronic Technologies, TU Dresden, Nöthnitzer Str. 61, Dresden, 01187 Sachsen, Germany

**Experimental section**

*Chemicals.* Ruthenium chloride hydrate (RuCl_3_·xH_2_O, ≥ 99.9%), Tri-ethylene glycol (TEG, 99%), Polyvinylpyrrolidone ((C_6_H_9_NO)_n_, M_w_ ~55000), Sodium borohydride (NaBH_4_, ≥ 98%), Chromium chloride Hexahydrate (CrCl_3_·6H_2_O, ≥ 98%), Deuterium oxide (D_2_O, 99.9 atom % D), Potassium hydroxide (KOH, ≥ 90%), and ethanol (C_2_H_6_O, ≥ 96%) were purchased from Sigma Aldrich.

*Synthesis of hcp Ru aerogels.* The hcp Ru aerogel was synthesized via a one-step reduction method at room temperature. First, 0.5 mL of RuCl_3_ solution (1 mg/mL) was added to 4.5 mL of deionized water and mixed under magnetic stirring for 3 minutes. Subsequently, 100 µL of NaBH_4_ solution (1 mM) was rapidly introduced, followed by gentle stirring for 10 seconds. The solution was then placed overnight, leading to the formation of a black hydrogel. The product was sequentially washed three times with deionized water and ethanol to remove residual impurities. Finally, freeze-drying was performed to obtain the final hcp Ru aerogel. In order to accurately determine the synthesis yield of aerogel, the amount of all precursors was increased tenfold in proportion during the experiment, and the final yield of hcp Ru was 90%.

*Synthesis of fcc Ru aerogels.* The fcc Ru aerogel was synthesized via a chemical reduction method. Briefly, 100 mg of polyvinylpyrrolidone was dissolved in 8 mL of tri-ethylene glycol (TEG), and the solution was heated to 200 °C, maintaining this temperature for 30 minutes. Subsequently, 45 mg of RuCl_3_ was dissolved in 4 mL of TEG and rapidly added to the preheated PVP-TEG solution, followed by a reaction at 200 °C for 3 h. After the reaction, the solution was cooled to room temperature, and twice its volume of acetone was added, shaking the mixture until it turned turbid. The mixture was then centrifuged at 6000 r/min for 10 minutes to collect the precipitate. The obtained precipitate was redispersed in 5 mL of deionized water and ultrasonicated for uniform dispersion. Then, 100 mg of NaBH_4_ was added, and the solution was placed overnight to allow precipitation. The product was subsequently washed three times with deionized water and ethanol to remove residual impurities. Finally, freeze-drying was performed to obtain the fcc Ru aerogel. The final yield of fcc Ru aerogel was 92.5%.

*Synthesis of fcc Ru nanoparticles.* The fcc Ru nanoparticles were synthesized via a chemical reduction method. Briefly, 100 mg of polyvinylpyrrolidone was dissolved in 8 mL of tri-ethylene glycol (TEG), and the solution was heated to 200 °C, maintaining this temperature for 30 minutes. Subsequently, 45 mg of RuCl_3_ was dissolved in 4 mL of TEG and rapidly added to the preheated PVP-TEG solution, followed by a reaction at 200 °C for 2.5 h. After the reaction, the solution was cooled to room temperature, and twice its volume of acetone was added, shaking the mixture until it turned turbid. The mixture was then centrifuged at 6000 r/min for 10 minutes to collect the precipitate. Then, the precipitate was washed three times with a mixture of acetone and ethanol (acetone/ethanol = 3:1, v/v). Finally, the product was dried at 60 ℃ for 12 h. The final yield of fcc Ru nanoparticles was 90.3%.

*Synthesis of fcc/hcp Ru aerogels.* The fcc/hcp Ru aerogel was also synthesized via a one-step reduction method at room temperature. First, five different concentrations of RuCl_3_ solutions were prepared by adding 0.5 mL of RuCl_3_ solution (1 mg/mL) to 0.5 mL, 1.5 mL, 2.5 mL, 3.5 mL, and 4.5 mL of deionized water, respectively, to obtain precursor solutions of varying concentrations. Subsequently, 100 µL of NaBH_4_ solution (1 mM) was quickly added to each solution and gentle stirred for 10 seconds, followed by static incubation for 1 hour to allow the gradual formation of black hydrogel. The resulting products were then sequentially washed three times with deionized water and ethanol to remove residual impurities. Finally, freeze-drying was performed to obtain fcc/hcp Ru aerogels with different phase ratios. In order to accurately determine the synthesis yield of aerogel, the amount of all precursors was increased tenfold in proportion during the experiment, and the final yield of fcc/hcp Ru was 91.4%.

*Synthesis of Cr doped fcc/hcp Ru aerogels.* The Cr-doped fcc/hcp Ru aerogel was also synthesized via a one-step reduction method at room temperature. First, a precursor solution was prepared by adding 0.5 mL of RuCl_3_ solution (1 mg/mL) to 0.5 mL of deionized water. Then, 5 µL, 10 µL, 15 µL, and 20 µL of CrCl_3_ solution (1 mg/mL) were separately added and stirred for 10 min. Subsequently, 100 µL of NaBH_4_ solution (1 mM) was quickly introduced into each mixed solution and gentle stirred for 10 seconds, followed by static incubation for 1 h to allow the gradual formation of black hydrogel. The resulting products were then sequentially washed three times with deionized water and ethanol to remove residual impurities. Finally, freeze-drying was performed to obtain fcc/hcp Ru aerogels with different Cr doping ratios. In order to accurately determine the synthesis yield of aerogel, the amount of all precursors was increased tenfold in proportion during the experiment, and the final yield of Cr_0.008_Ru_0.992 fcc/hcp_, Cr_0.022_Ru_0.978 fcc/hcp_, Cr_0.033_Ru_0.967 fcc/hcp_, and Cr_0.041_Ru_0.959 fcc/hcp_ aerogels was measured to be 93.1%, 94.4%, 93.4%, and 91.1%, respectively.

*Material Characterizations.* The crystalline structure of the catalyst was characterized by X-ray diffraction (XRD) using a Bruker AXS D2 PHASER equipped with Cu-Kα radiation operated with a scan rate of 0.3 °/min. X-ray photoemission spectroscopy (XPS) measurements were carried out on an ESCALAB 250Xi by Thermo Scientific in an ultrahigh vacuum chamber (base pressure: 2 × 10^−10^ mbar) with an XR6 monochromated Al Kα X-ray source (hν = 1486.6 eV) and a pass energy of 20 eV. UPS measurements were conducted with double differentially pumped He discharge lamp (hν = 21.22 eV) with a pass energy of 2 eV and a bias of −10 V. Raman spectroscopy was performed using a high-resolution confocal Raman microscope (MonoVista CRD+, S&I Spectroscopy & Imaging GmbH) equipped with a liquid-nitrogen-cooled CCD PyLoN: 100 BRX detector (Princeton Instruments) and a 514 nm laser source. The chemical composition of the catalyst was analyzed by inductively coupled plasma atomic emission spectroscopy (ICP-AES) using an iCAP 7400 system (Thermo Scientific). Transmission electron microscopy (TEM) images were recorded using a JEOL JEM 1400 plus equipped with a LaB_6_ cathode at an accelerating voltage of 120 kV. The high-resolution TEM images were acquired by means of a TF Titan^3^ 80-300 double aberration-corrected transmission electron microscope operating at 300kV acceleration voltage equipped with a Gatan OneView camera. Image processing was performed by Gatan Digital Micrograph. EDX spectra were recorded in the same instrument by means of AMETEK EDAX TOPS 300 ST spectrometer using a screen current of 11nA and a probe diameter of 1.5µm. Local STEM-EELS measurements were conducted by means of the Hitachi HF3300S transmission electron microscope operating at an acceleration voltage of 300 kV equipped with a CEOS CEFID energy filter and Quantum Detectors Merlin 4R direct electron detector using a convergence semi-angle of 21.5 mrad and collection semi-angle of 20 mrad and energy range of 91 eV at the EELS detection plane. Nitrogen physisorption isotherms were measured at 77 K on an AUTOSORB-iQ-C-XR from Quantachrome^®^. The specific surface area was determined using the Brunauer-Emmett-Teller (BET) method and the pore size distribution was determined from the isotherm using density functional theory (DFT) method.

*Electrochemical measurements.* A standard three-electrode system coupled with an electrochemical work station was used for evaluating the electrocatalytic activity. Hg/HgO, Pt foil and glassy carbon electrode (GCE) with a diameter of 3 mm, were used as the reference electrode, counter electrode and working electrode, respectively. The electrocatalyst ink was prepared by dispersing 1 mg catalysts in a 0.2 ml ethanol/H_2_O/5% Nafion solution (0.48:0.5:0.02) mixture. Then, 10 μl of the ink was dropped onto the GCE electrode and dried naturally. To get the real potential of the samples, all electrochemical measurements underwent an iR-correction, where i represents the test current and R denotes the solution resistance. All measurements were adjusted to the reversed hydrogen electrode using the equation^[1]^:

$$E_{\mathrm{RHE}}= E_{Hg/HgO}+0.059pH+0.098V$$

*Calculation of electrochemically active surface area (ECSA).* The ECSA was calculated using the formula^[2]^:

$$\mathrm{ECSA}= \frac{C_{\mathrm{dl}}}{C_{s}}$$

where C_s_ is the specific capacitance of a perfectly smooth metal electrode (C_s_ = 0.04 mF cm^-2^). The electrochemical double-layer capacitance (C_dl_) was determined by plotting the difference between the anodic and cathodic current densities (Δ*j* = *di-dj*) against various scan rates.

*Calculation of turnover frequency (TOF).* The TOF was calculated according to the following equation^[3]^:

$$\mathrm{TOF}= \frac{I}{2aF}$$

Where I is the current of the polarization curves. F is the Faraday constant (96485 C mol^-1^). 2 represent the electrons consumed to form H_2_ molecules from water. a represents the mole number of metal ions. The mass of metal can be obtained from the ICP-OES.

*CO stripping test.* CO adsorption was firstly performed with i-t test at 0.05 V versus reversible hydrogen electrode for 10 min in a CO saturated 0.1 M KOH aqueous solution. The CO stripping current was obtained by CV in the potential range of 0-1 V versus RHE at a scan rate of 20 mV s^-1^.

*Calculation of the Faradaic efficiency.* The generated H_2_ at the cathode was collected using the drainage method. At a current density of -50 mA cm^-2^, the volume of H_2_ was recorded every 5 minutes. The theoretical H_2_ production was calculated based on the assumption that 100% of the current was used for the OER. The theoretical H_2_ amount (N_1_, in mol) was determined using the formula^[4]^:

$N_{1}= \frac{Q}{\mathrm{nF}}$​

where Q = I × t is the total charge (I represents the current, obtained by multiplying the current density by the the electrode surface area, and t is the time in seconds), F is the Faraday constant (96485 C mol^-1^), and n = 2 is the number of electrons transferred per H_2_ molecule.

The actual H_2_ amount (N_2_, in mol) was calculated using the formula:

$$N_{2}=\frac{V}{V_{m}}$$

where V is the volume of H_2_ produced, and V_m_ is the molar volume of H_2_ (22.4 L mol^-1^).

The Faradaic efficiency (FE) for hydrogen production was then determined using the equation^[5]^:

$$FE=\frac{2VF}{\mathrm{It}V_{m}} \times100\%$$

*Density functional theory calculation.* Based on density-functional theory^[6]^ (DFT), the first-principles calculations were performed in the present work by using the Vienna ab initio Simulation Package (VASP).^[7]^ As for the exchange–correlation potential, we adopt the generalized gradient approximation (GGA) of the Perdew−Burke−Ernzerhof (PBE) method.^[8]^ In this calculation, the cutoff energy was set as 400 eV, and a Γ-centered k-point mesh of 3×3×1 in the BZ was used. For the crystal structure of the slab model, a vacuum space larger than 20 Å was built to avoid interactions between layers. The long-range van der Waals interactions were considered using the DFT-D2 method.^[9]^ For the calculation of the ΔG_H*_ and ΔE_OH-_ of hydrogen adsorption, the convergence threshold was set to be 10^-4^ eV for energy and 0.01 eV Å^-1^ for force. The ΔG_H*_ and ΔE_OH_ values were obtained based on the 3 × 3 supercell model.


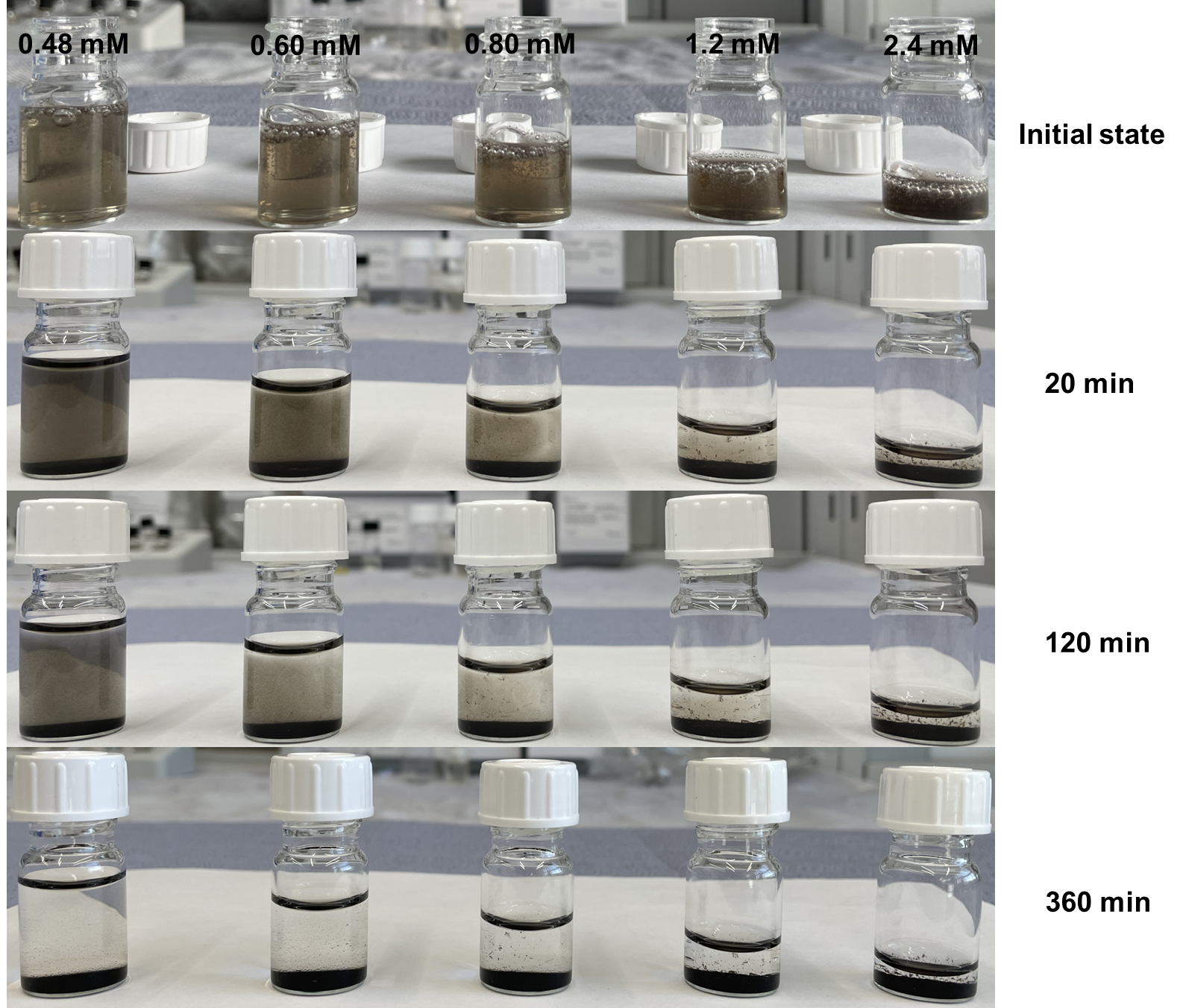


**Figure S1.** Photographs of the process of preparing Ru aerogels using RuCl_3_ precursors with different concentrations.


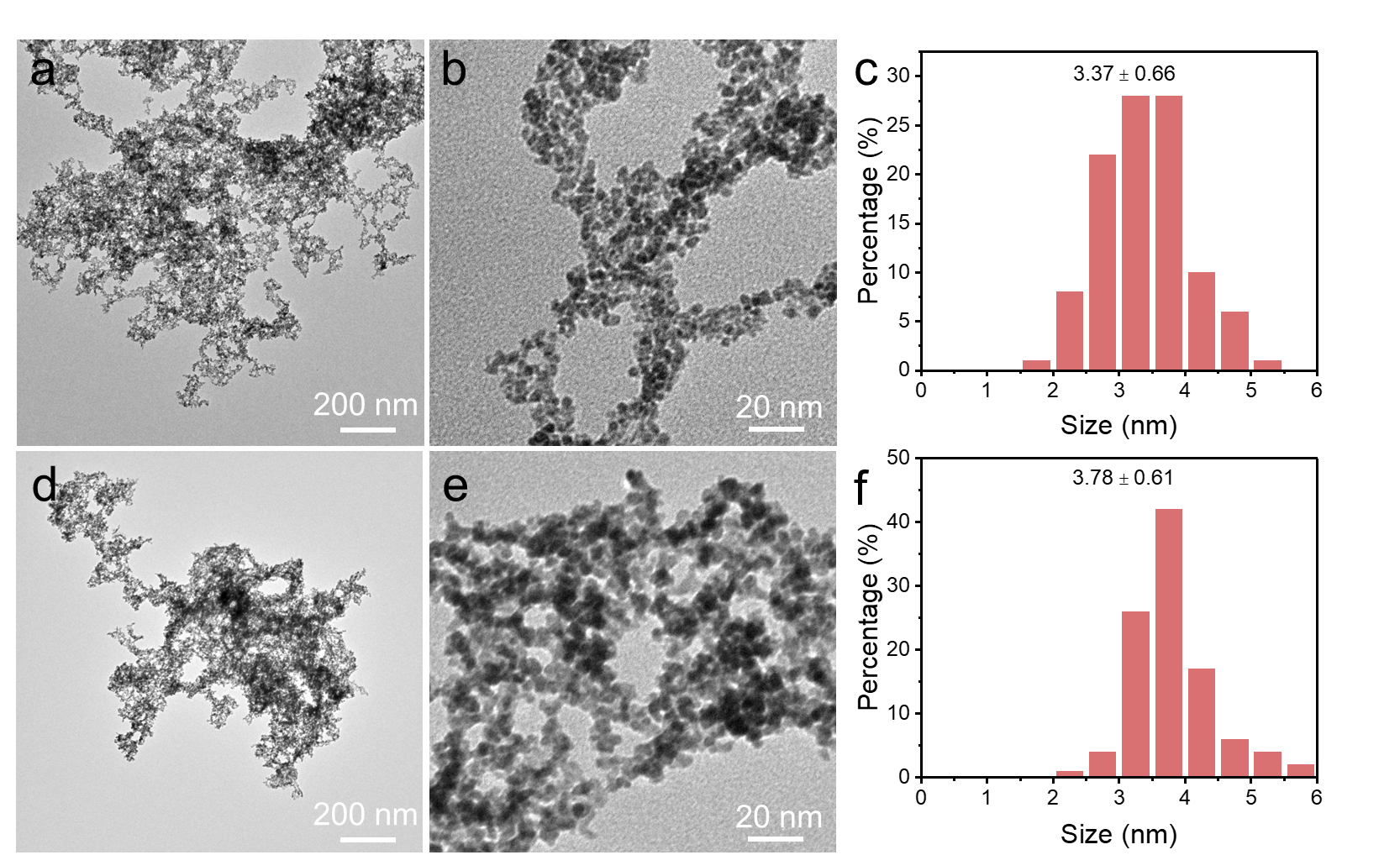


**Figure S2**. TEM images and size distributions of (a-c) Ru aerogel with RuCl_3_ concentration of 0.48 mM, and (d-f) Ru aerogel with RuCl_3_ concentration of 2.40 mM.


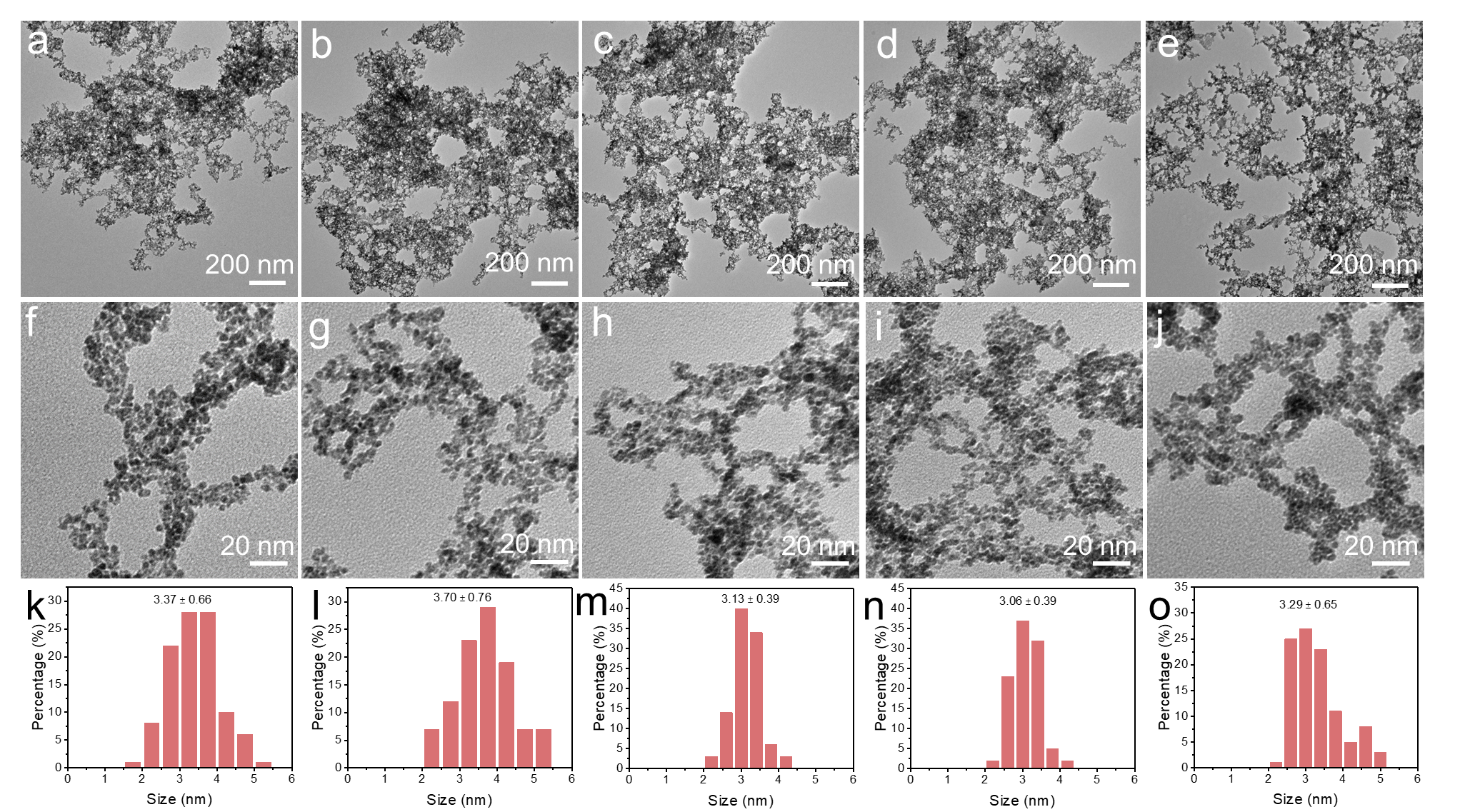


**Figure S3.** TEM images and size distributions of (a, f, k) Ru_fcc/hcp_; (b, g, l) Cr_0.002_Ru_0.998 hcp/fcc_; (c, h, m) Cr_0.022_Ru_0.978 hcp/fcc_; (d, i, n) Cr_0.033_Ru_0.967 hcp/fcc_; (e, j, o) Cr_0.041_Ru_0.959 fcc/hcp_.


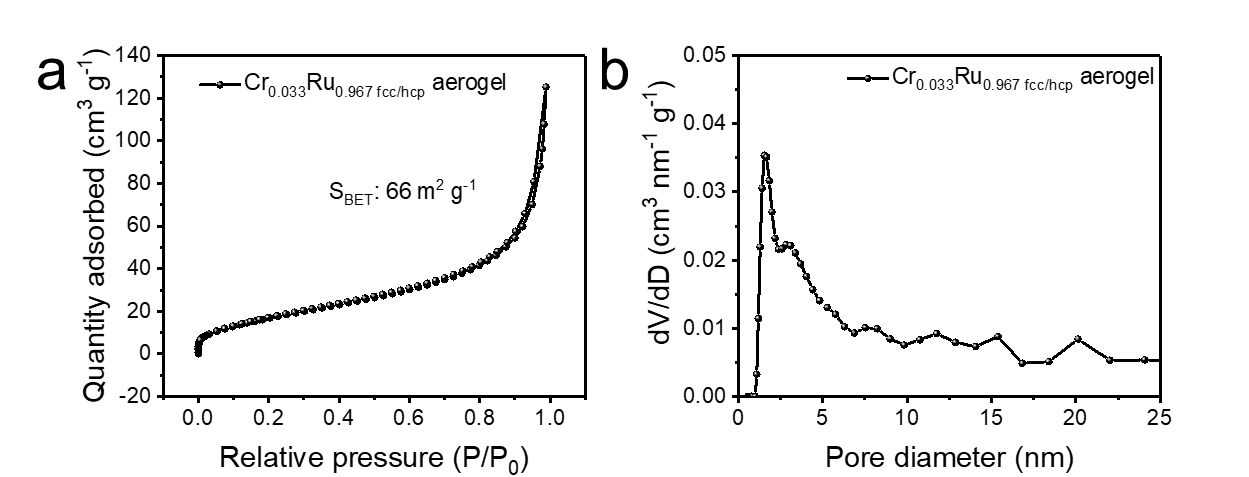


**Figure S4.** (a) N_2_ physisorption isotherms and (b) pore size distribution of the Cr_0.033_Ru_0.967 fcc/hcp_ aerogel.


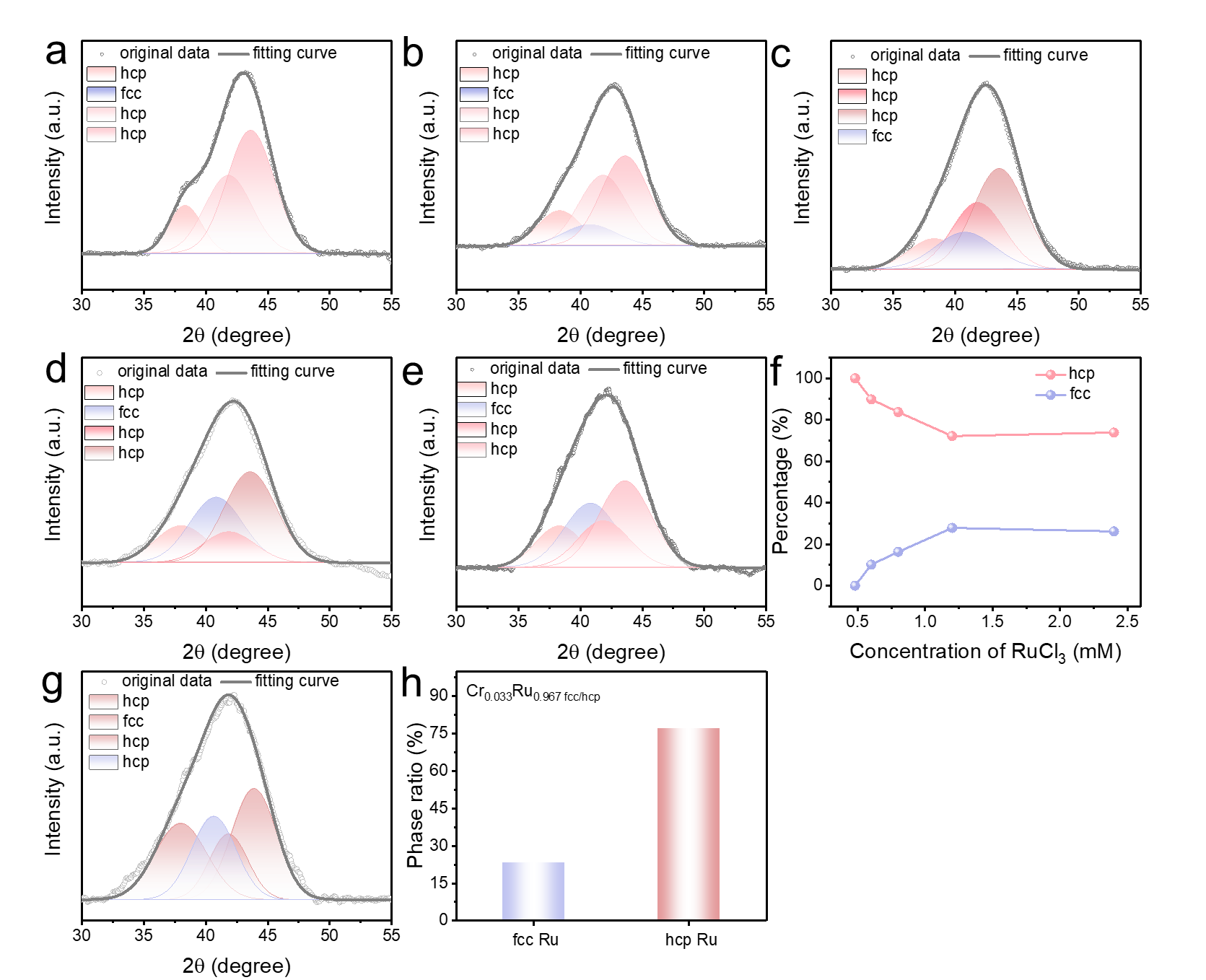


**Figure S5**. (a-e) PXRD patterns with the fitted profiles of Ru aerogels obtained at different concentrations (0.48, 0.60, 0.80, 1.20, and 2.40 mM) of the RuCl_3_ precursors. (f) The percentage of the fcc phase and the hcp phase in the Ru aerogels varies with the concentration of the RuCl_3_ precursors. (g) PXRD patterns with the fitted profiles of the Cr_0.033_Ru_0.967 fcc/hcp_ aerogels. (h) The percentage of the fcc phase and the hcp phase in the Cr_0.033_Ru_0.967 fcc/hcp_ aerogel.

The phase composition of Ru aerogels, specifically the relative amounts of hcp and fcc structures, was quantitatively analyzed using the reference intensity ratio method.^[10,11]^ This analytical method involves determining the ratio between the highest diffraction peak intensity of the target material (I) and that of a standard reference material (I_c_) in an equimass mixture. Corundum (α-Al_2_O_3_) is typically employed as the calibration standard due to its well-defined diffraction characteristics and chemical inertness. According to the ICDD crystallographic database, the values (I/I_c_) for the predominant reflections of Ru phases are as follows: the hcp phase exhibits a characteristic (101) reflection with a *d*-spacing of 2.05 Å (2θ = 43.62°), yielding an I/I_c_ of 15.29, while the fcc phase shows a primary (111) reflection at *d* = 2.21 Å (2θ = 40.86°) with an I/I_c_ of 16.44. The phase of Ru aerogels could be quantitatively determined using the equation:

$$\frac{I_{hcp}}{I_{fcc}}= \frac{(I/{I_{C})_{hcp}}X_{hcp}}{(I/{I_{C})_{fcc}}X_{fcc}}$$

Here, *I*_hcp_ and *I*_fcc_ are the intensities of Ru hcp (101) and fcc (111) peaks calculated from the fitted profiles of the obtained PXRD data (Figures 1b), and *X*_hcp_ and *X*_fcc_ are the fractions of hcp and fcc phases in the sample, respectively.


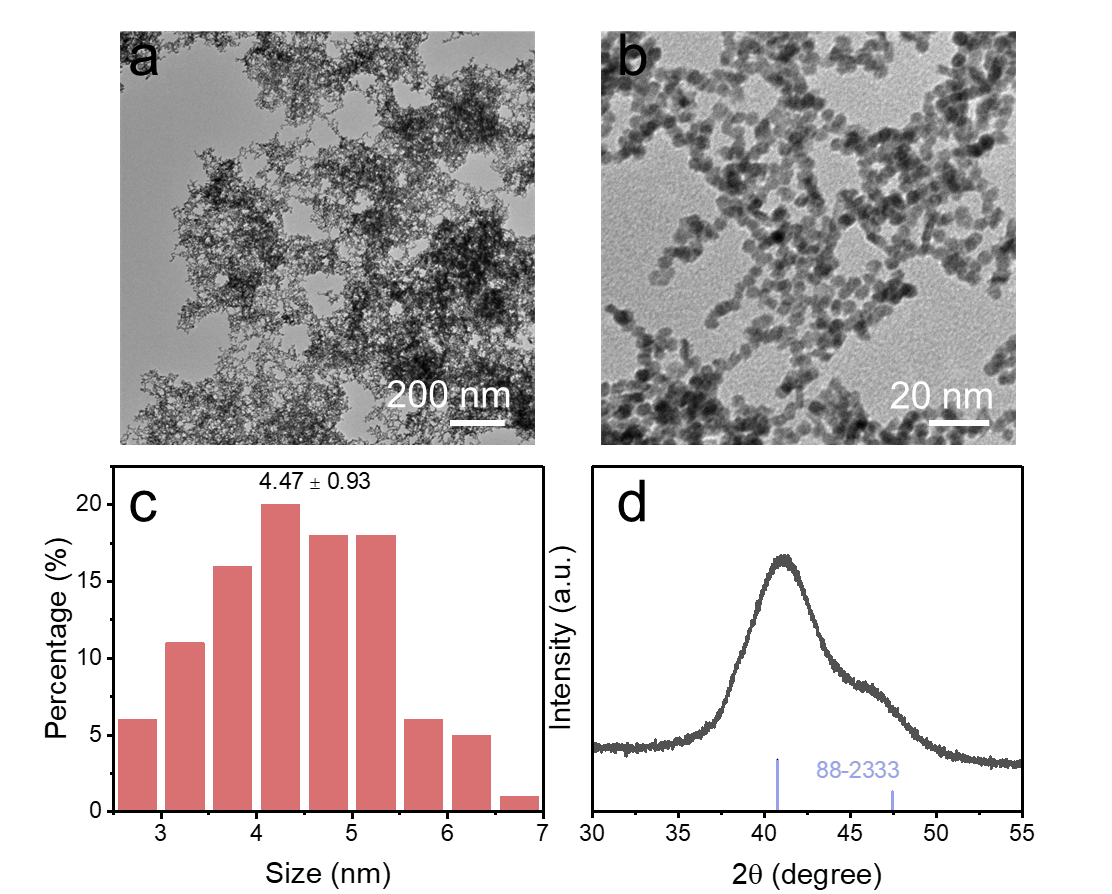


**Figure S6.** (a, b) TEM images of Ru_fcc_ aerogel. (c) Size distribution of Ru_fcc_ aerogel. (d) XRD pattern of Ru_fcc_ aerogel. The purple lines in Figure d represent standard fcc Ru (JCPDS No. 88-2333).


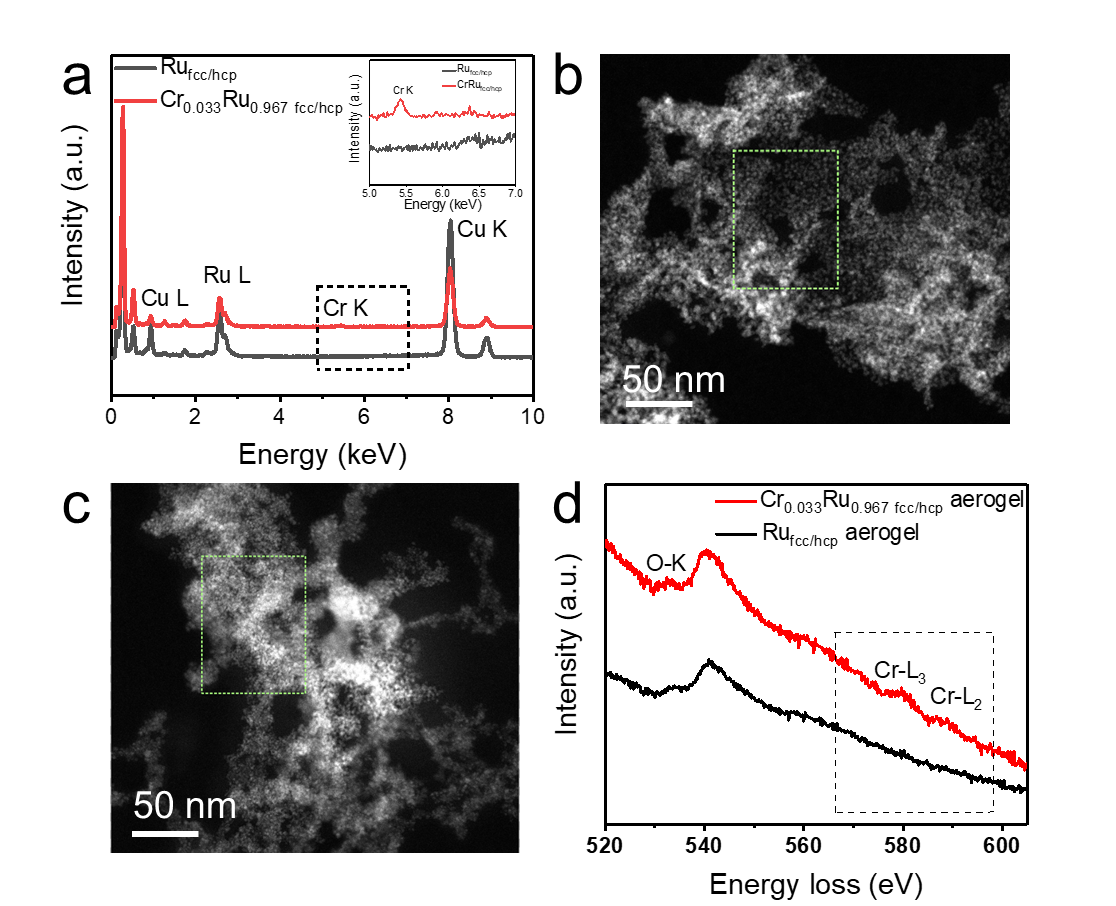


**Figure S7.** (a) EDX spectra of Ru_fcc/hcp_ and Cr_0.033_Ru_0.967 fcc/hcp_ aerogels, respectively. The Cu peaks in the EDX spectra originate predominantly from the copper content of the TEM holder and sample grid. HAADF images of (b) Ru_fcc/hcp_ and (c) Cr_0.033_Ru_0.967 fcc/hcp_ aerogels. The green dashed line boxes in (b) and (c) indicate the STEM-EELS scanning areas. (d) Electron energy loss spectra of Ru_fcc/hcp_ and Cr_0.033_Ru_0.967 fcc/hcp_ aerogels averaged within the respective scanning areas marked in (b) and (c).

**Figure S8.** Zeta potential of Ru-based aerogels.


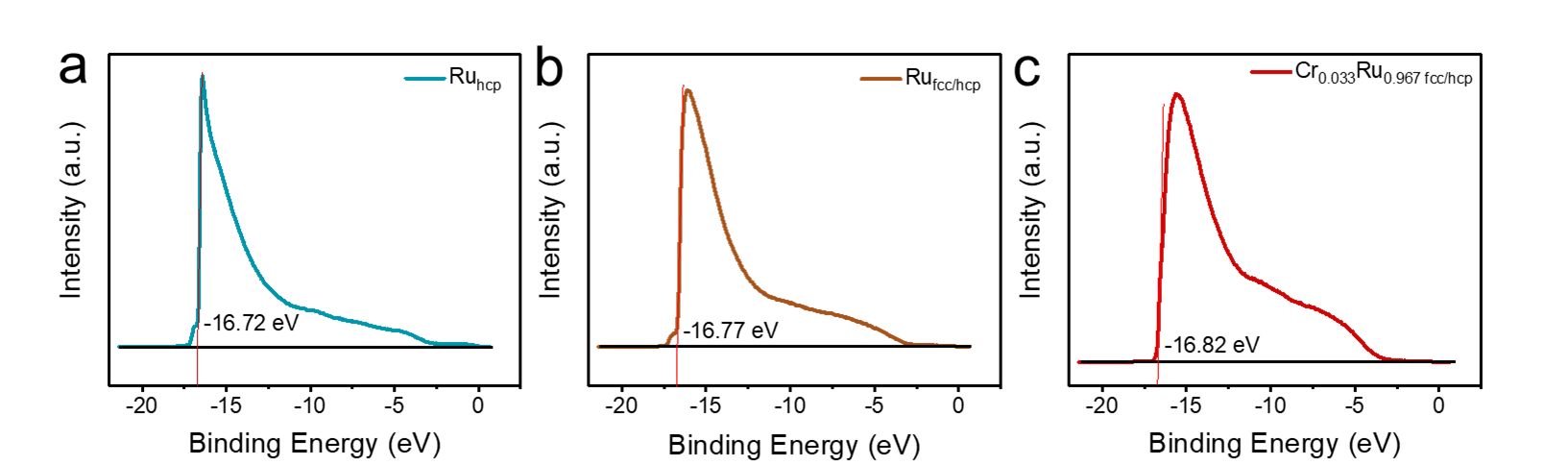


**Figure S9.** UPS spectra of Ru_hcp_, Ru_fcc/hcp_ and Cr_0.033_Ru_0.967 fcc/hcp_ aerogels.

The work function was calculated according to the equation of 𝑊 = ℎ𝑣 − 𝐸_cutoff_, where ℎ𝑣 is the incident photon energy (21.22 eV) and 𝐸_cutoff_ is the normalized secondary electron cutoff (**Figure S8**). Values of 4.5, 4.45 and 4.40 eV were determined for Ru_hcp_, Ru_fcc/hcp_ and Cr_0.033_Ru_0.967 fcc/hcp_ aerogels, respectively.


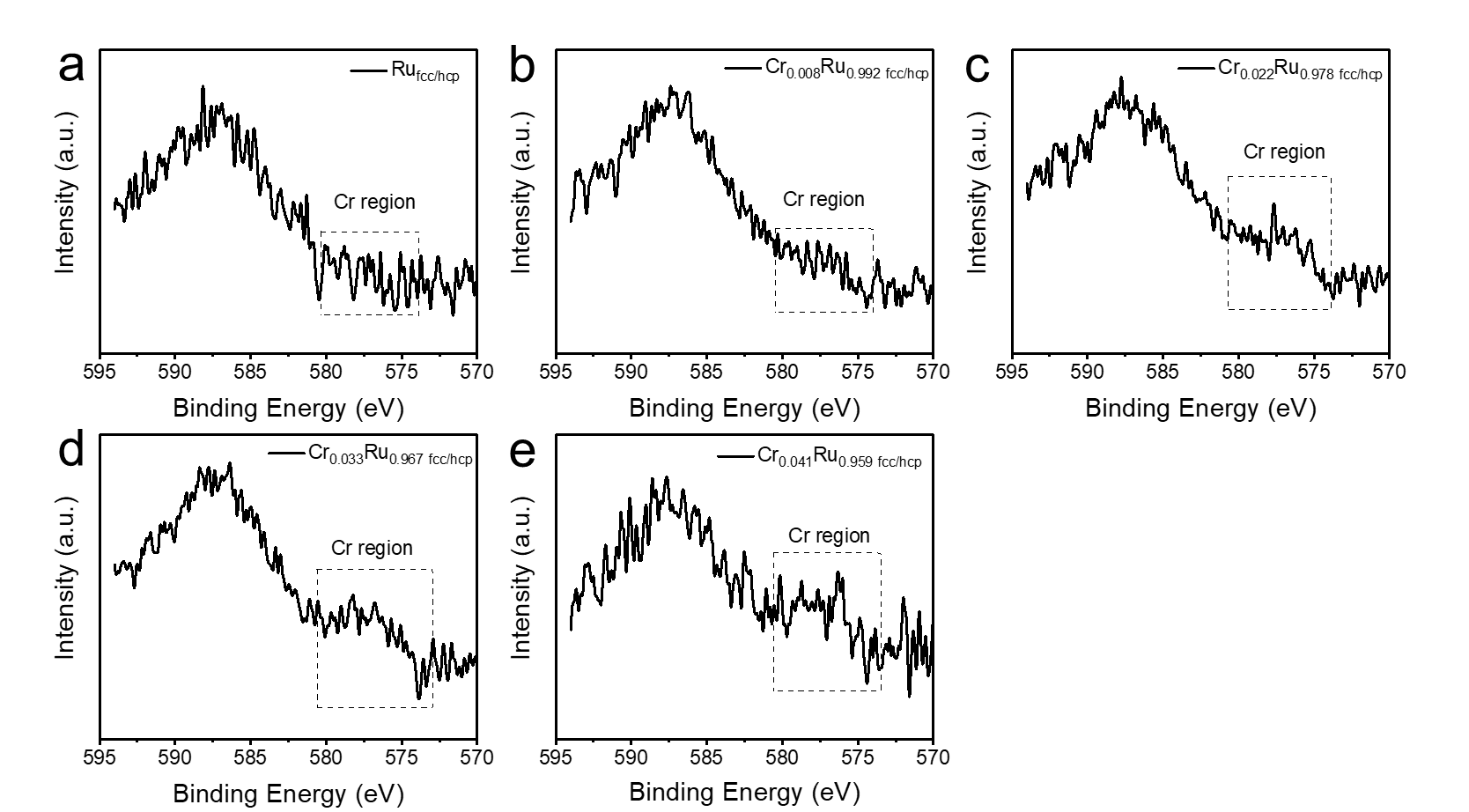


**Figure S10.** Cr 2p XPS spectra of (a) Ru_fcc/hcp_, (b) Cr_0.008_Ru_0.992 fcc/hcp_, (c) Cr_0.022_Ru_0.978 fcc/hcp_, (d) Cr_0.033_Ru_0.967 fcc/hcp_, and (e) Cr_0.041_Ru_0.959 fcc/hcp_ aerogels.


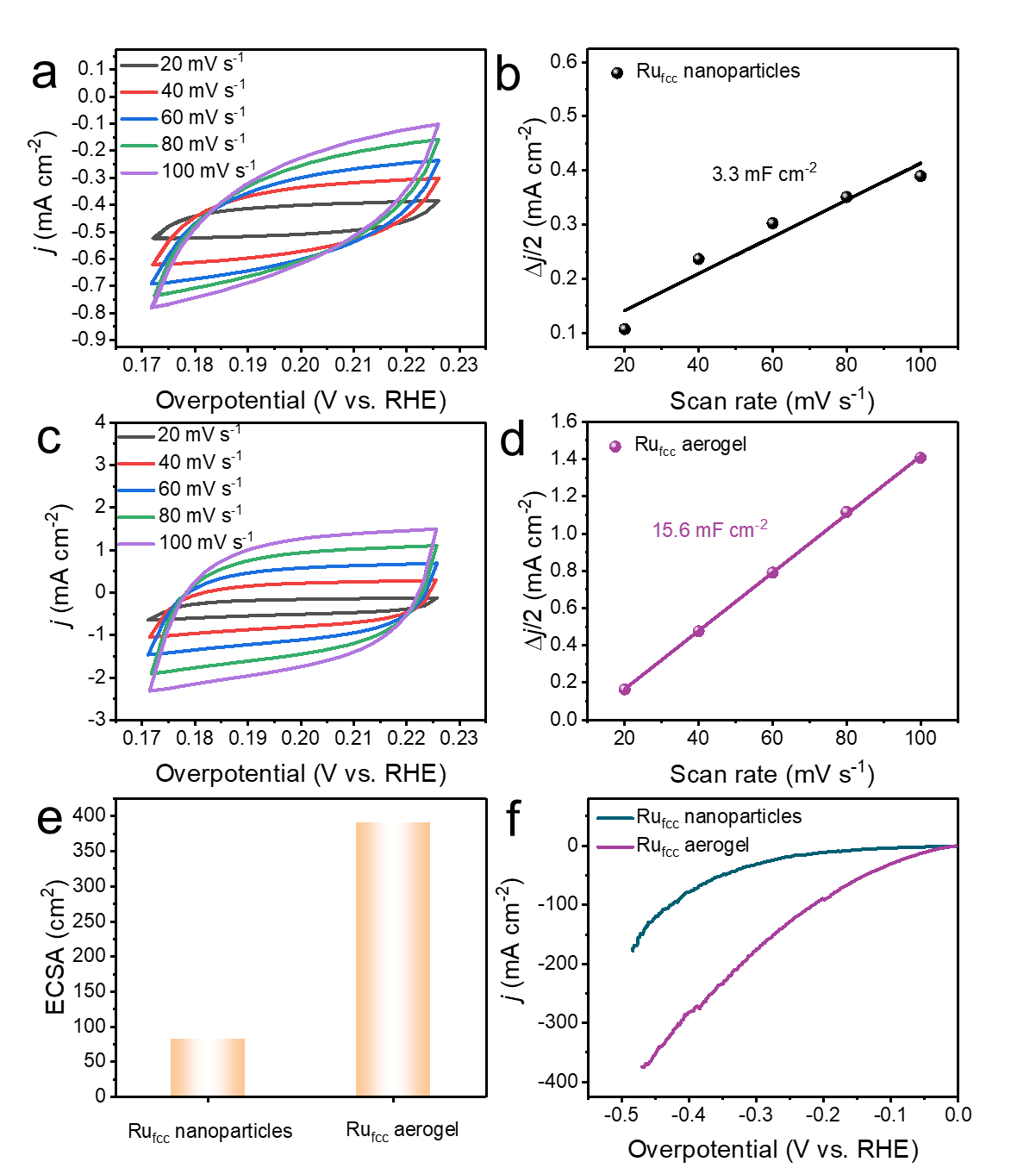


**Figure S11.** (a, c) CV curves, (b, d) C_dl_ values, (e) ECSA, and (f) polarization curves of Ru_fcc_ nanoparticles and Ru_fcc_ aerogel, respectively.


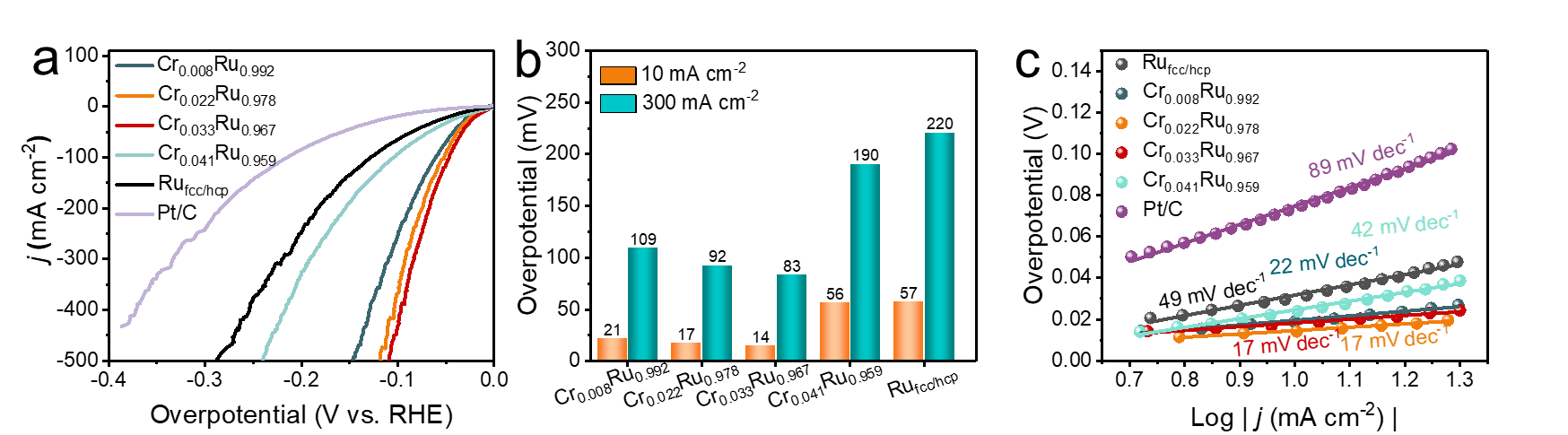


**Figure S12.** (a) Polarization curves Ru-based aerogels and Pt/C. (b) Comparison of overpotentials of Ru-based aerogels at 10 and 300 mA cm^-2^, respectively. (c) Tafel slope of Ru-based aerogels.


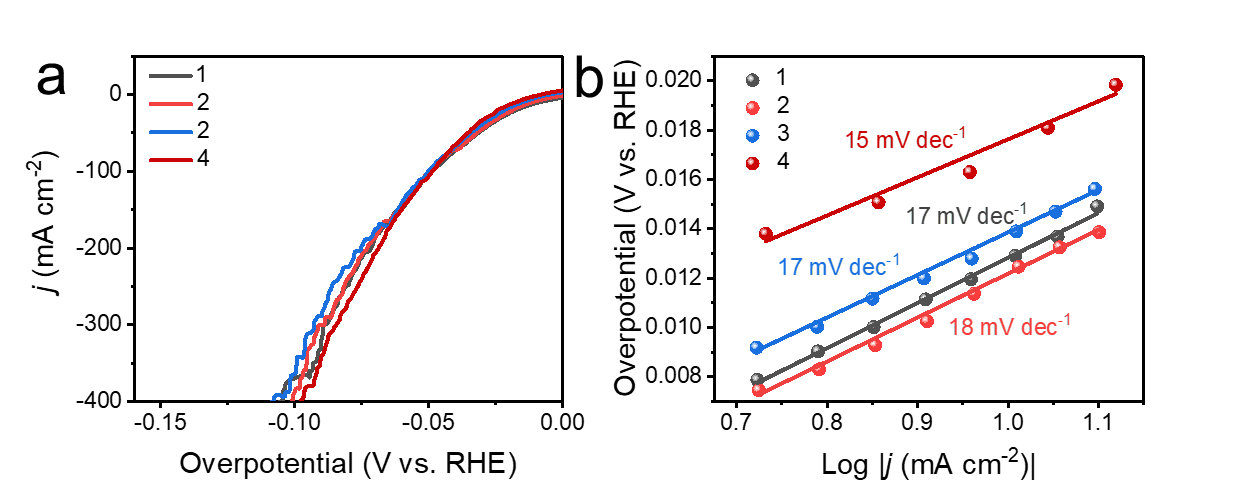


**Figure S13.** (a) Polarization curves and (b) Tafel plots of Cr_0.033_Ru_0.967 fcc/hcp_ aerogel measured from four independent experiments.


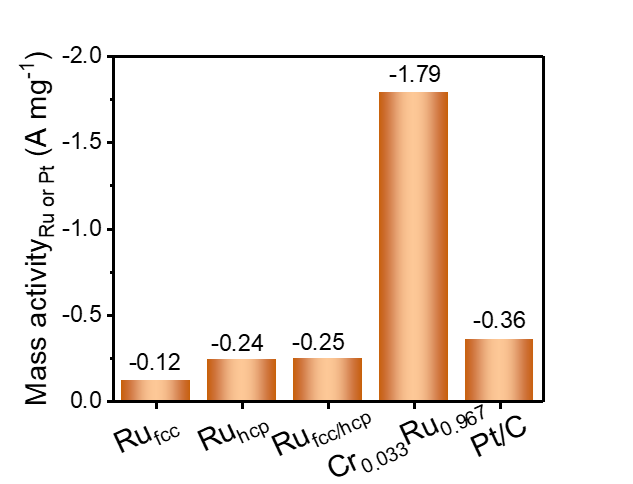


**Figure S14.** Mass activity of Ru-based aerogels and Pt/C.


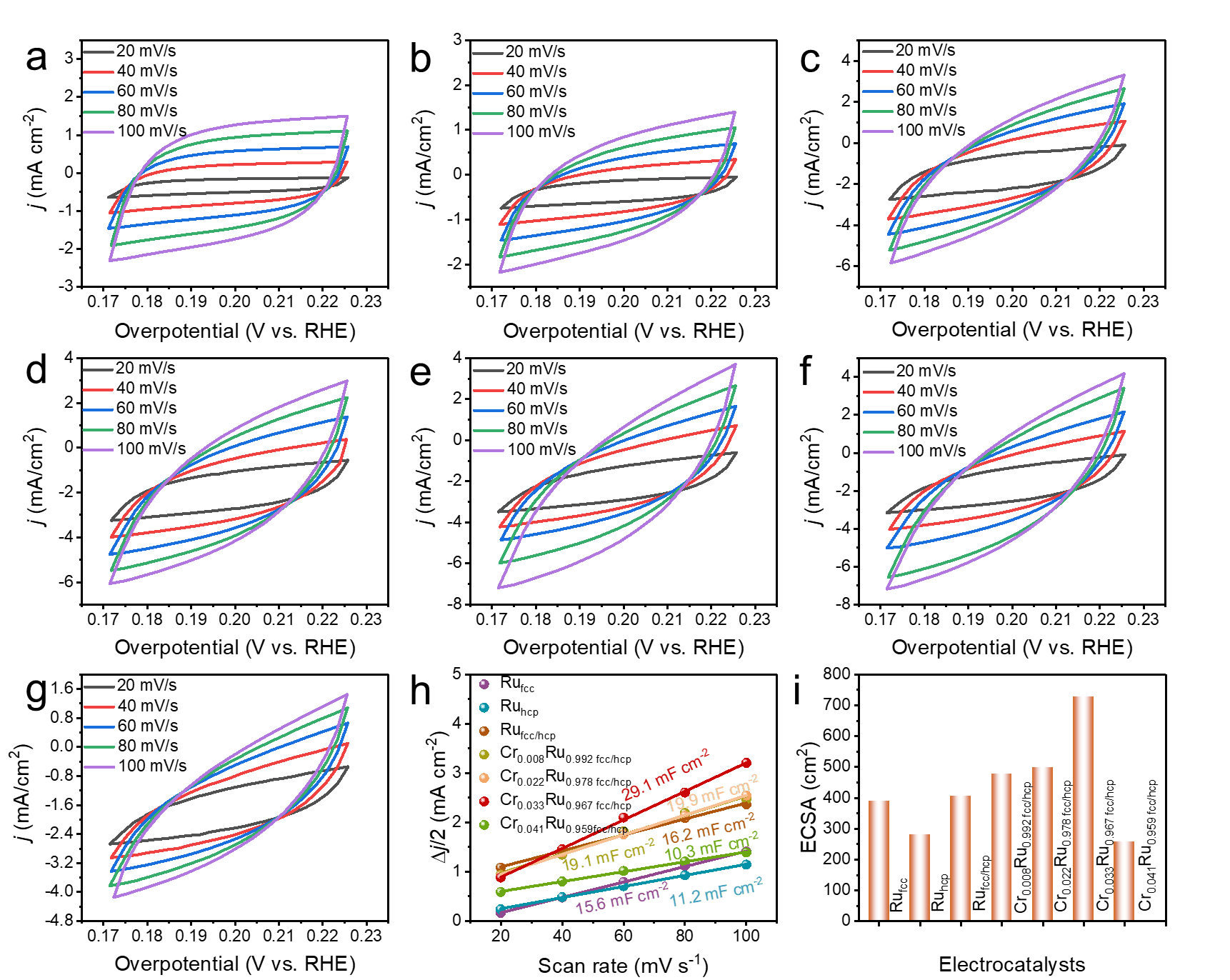


**Figure S15.** CV curves of (a) Ru_fcc_, (b) Ru_hcp_, (c) Ru_fcc/hcp_, (d) Cr_0.008_Ru_0.992_ _fcc/hcp_, (e) Cr_0.022_Ru_0.978_ _fcc/hcp_, (f) Cr_0.033_Ru_0.967_ _fcc/hcp_, and (g) Cr_0.041_Ru_0.959_ _fcc/hcp_. (h) C_dl_ values and (i) ECSA values of Ru-based aerogels.


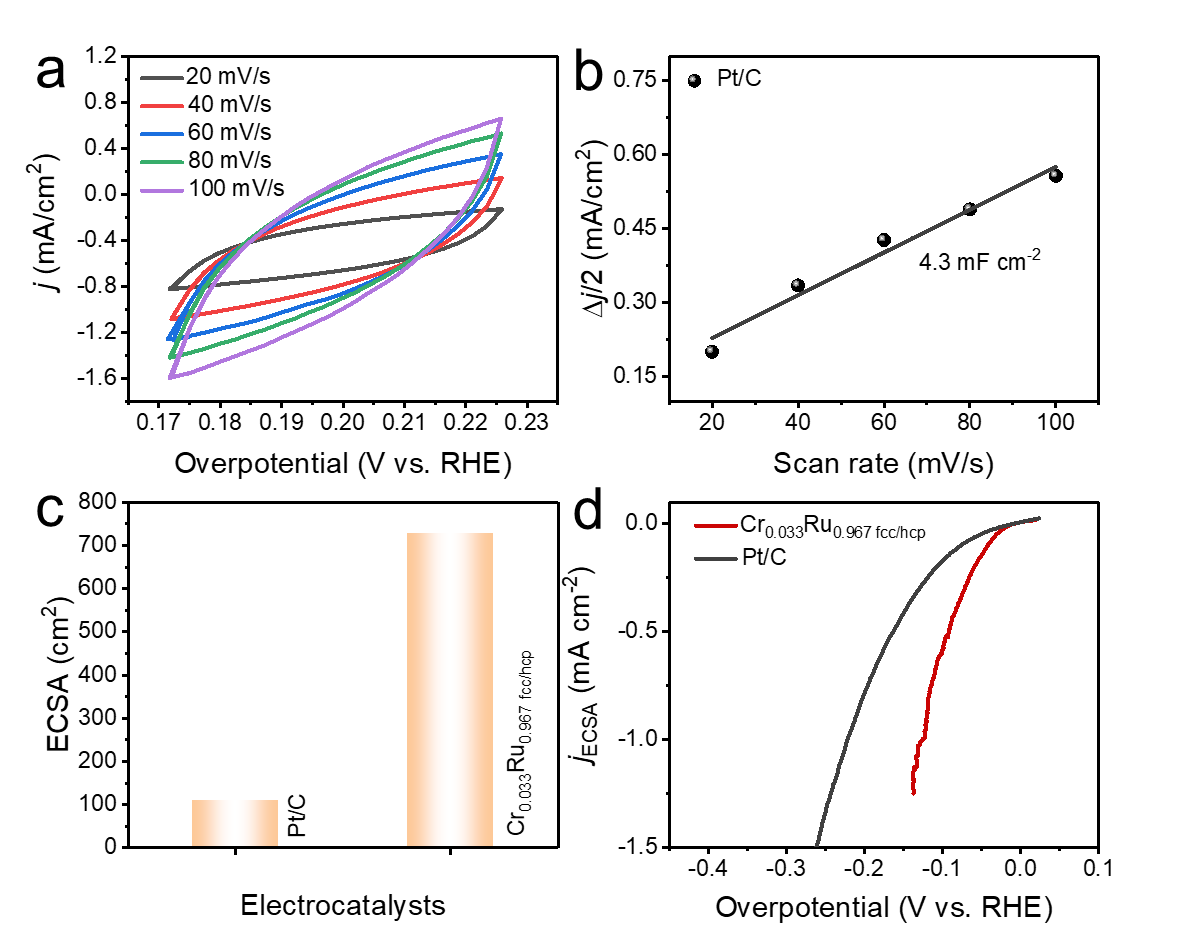


**Figure S16.** (a) CV curves and (b) C_dl_ value of Pt/C. (c) ECSA values and (d) Polarization curves normalized by ECSA of Cr_0.033_Ru_0.967 fcc/hcp_ aerogel and Pt/C.


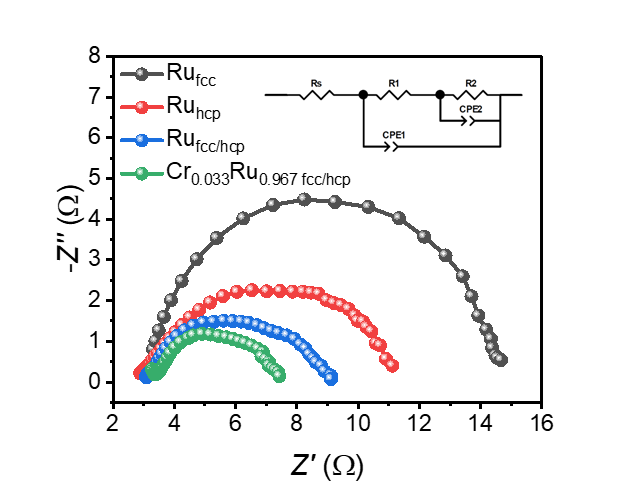


**Figure S17.** Nyquist plots of Ru-based aerogel.


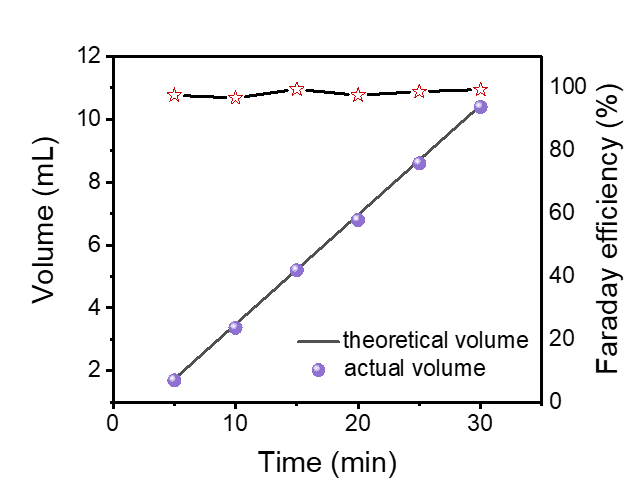


**Figure S18.** Faraday efficiency of Cr_0.033_Ru_0.967 fcc/hcp_ for the HER.


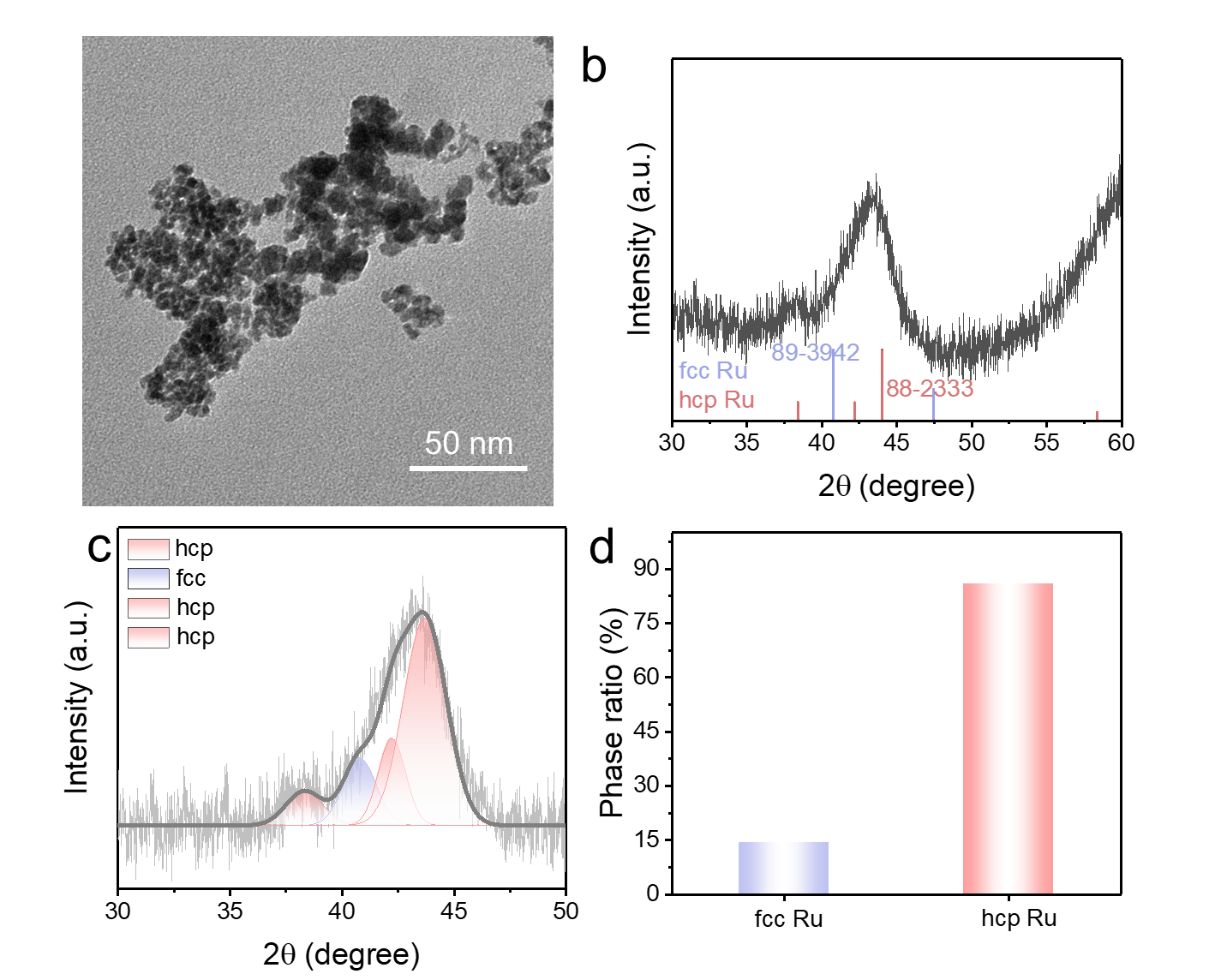


**Figure S19.** (a) TEM image and (b) XRD pattern of Cr_0.033_Ru_0.967 fcc/hcp_ aerogel after the stability test. (c) XRD diffraction peaks and crystal phase fitting based on b. (d) Phase ratio of Cr_0.033_Ru_0.967 fcc/hcp_ aerogel after the stability test.


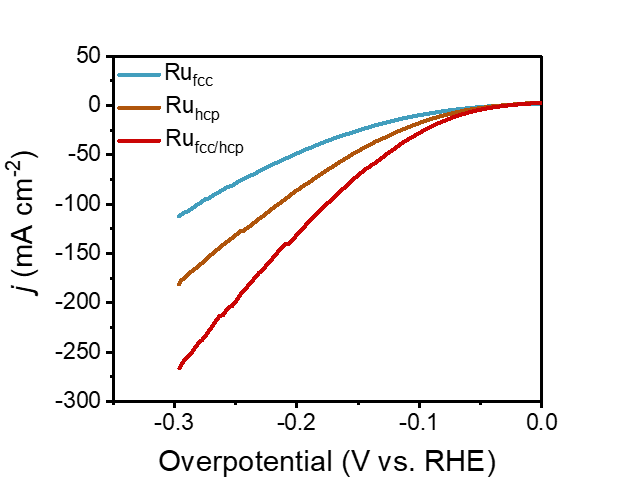


**Figure S20.** Polarization curves of Ru_fcc_, Ru_hcp_, and Ru_fcc/hcp_ in 0.5 M H_2_SO_4_.

**Figure S21.** CV curves of Ru_fcc/hcp_ and Cr_0.041_Ru_0.959_ _fcc/hcp_ aerogels.


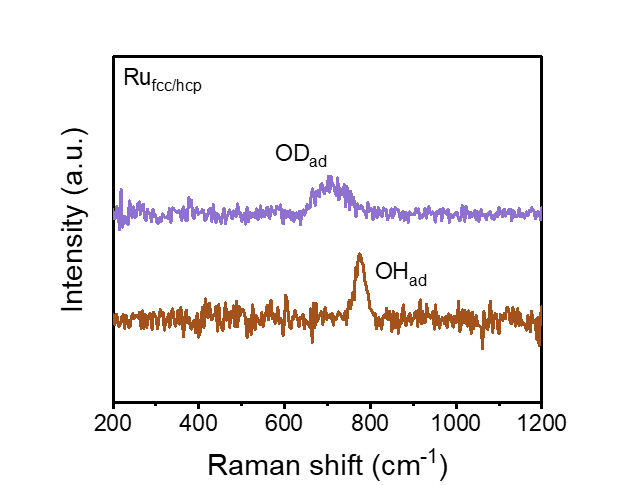


**Figure S22.** Raman spectroscopy of Ru_fcc/hcp_ aerogel in KOH/H_2_O solution and KOD/D_2_O solution.


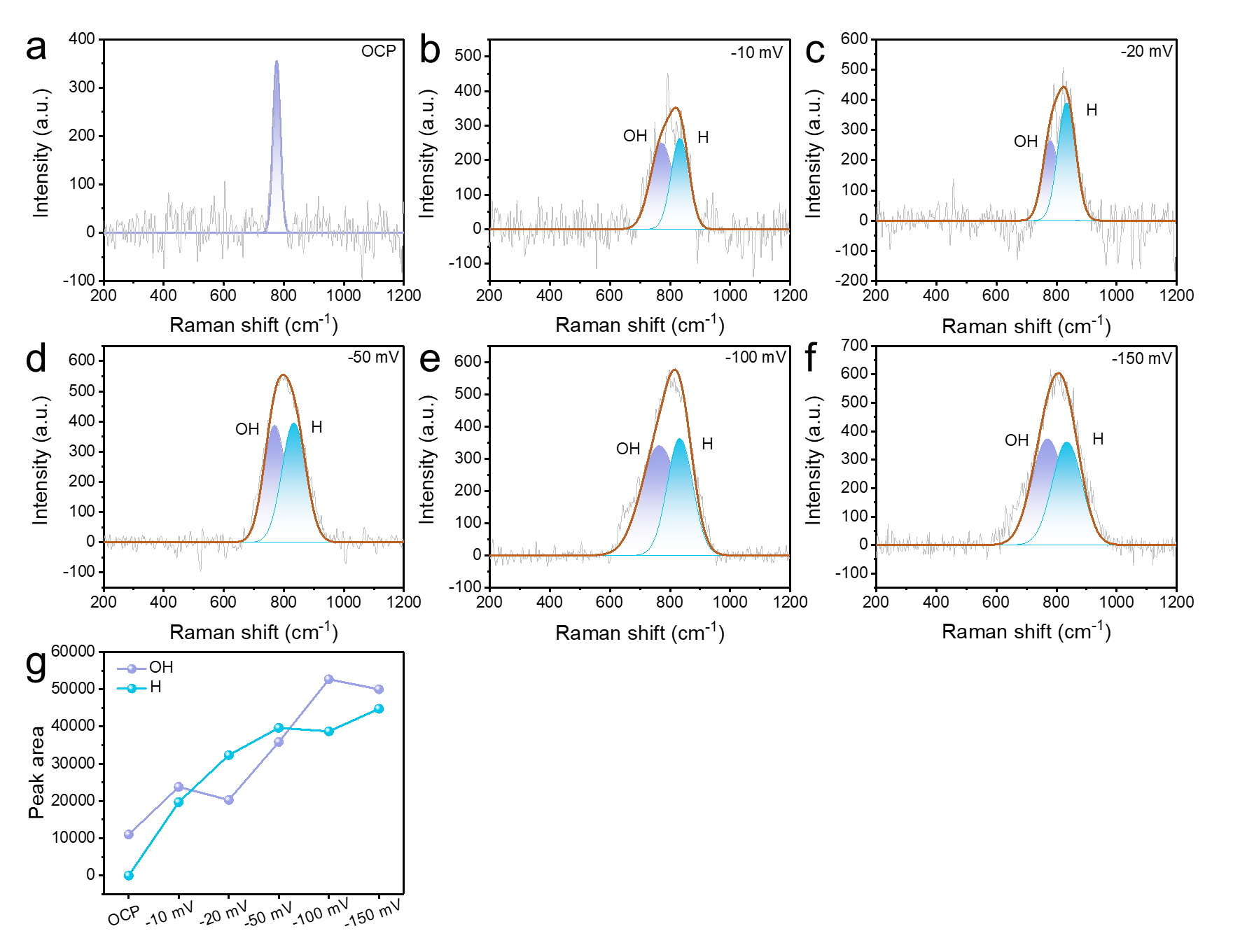


**Figure S23.** (a-f) Raman spectra of Ru_fcc/hcp_ aerogel under different applied potentials and fitting of OH and H characteristic peaks. (g) The change of the peak area for OH and H at different applied potentials.


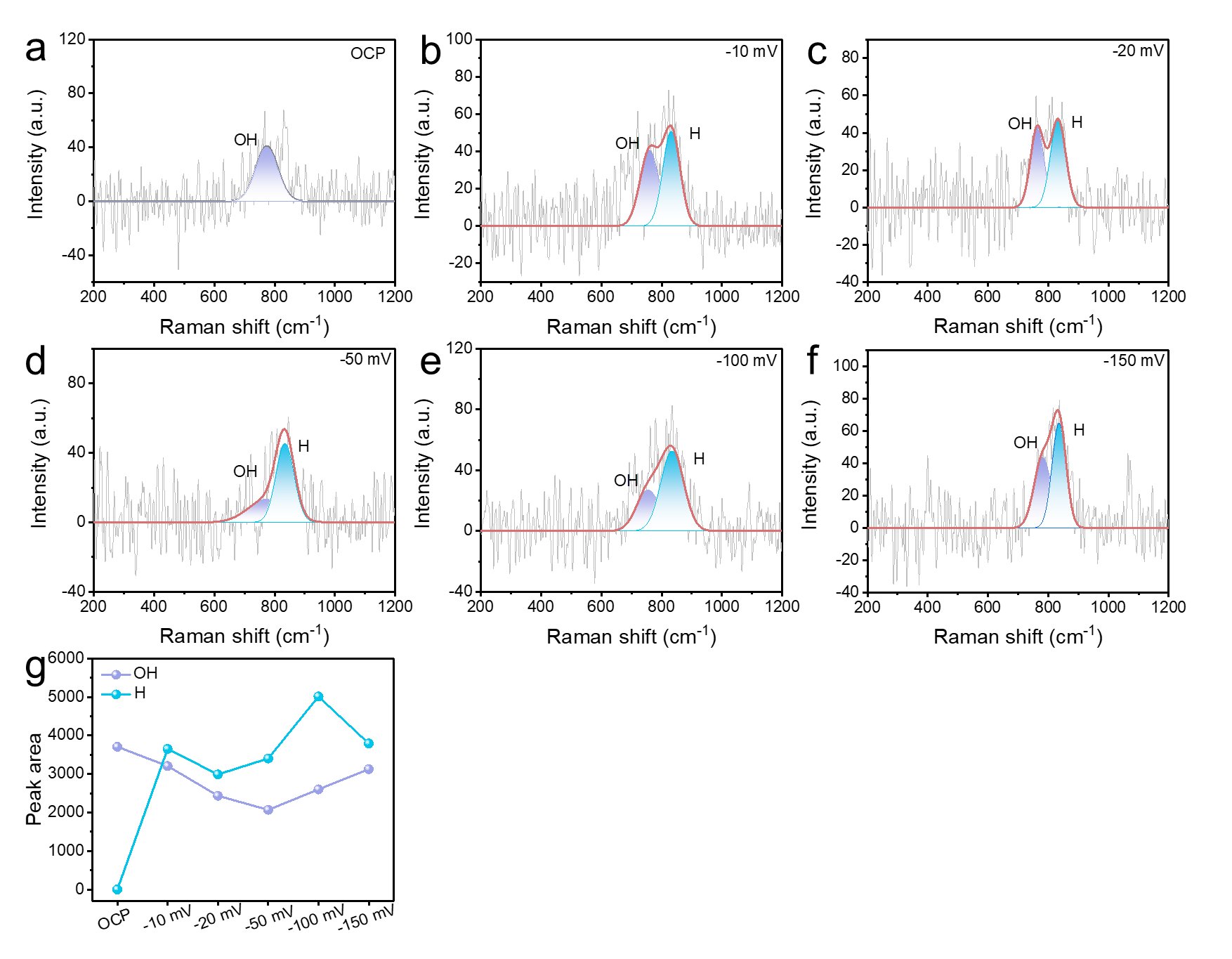


**Figure S24.** (a-f) Raman spectra of Cr_0.033_Ru_0.967_ _fcc/hcp_ aerogel under different applied potentials and fitting of OH and H characteristic peaks. (g) The change of the peak area for OH and H at different applied potentials.


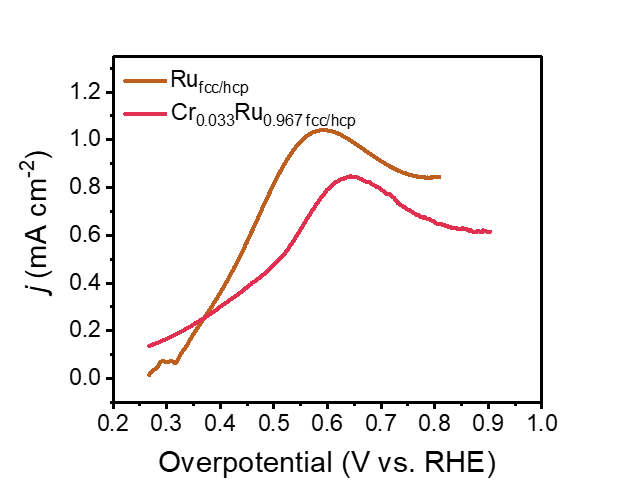


**Figure S25.** CO stripping experiment of Ru_fcc/hcp_ and Cr_0.033_Ru_0.967 fcc/hcp_ aerogels.


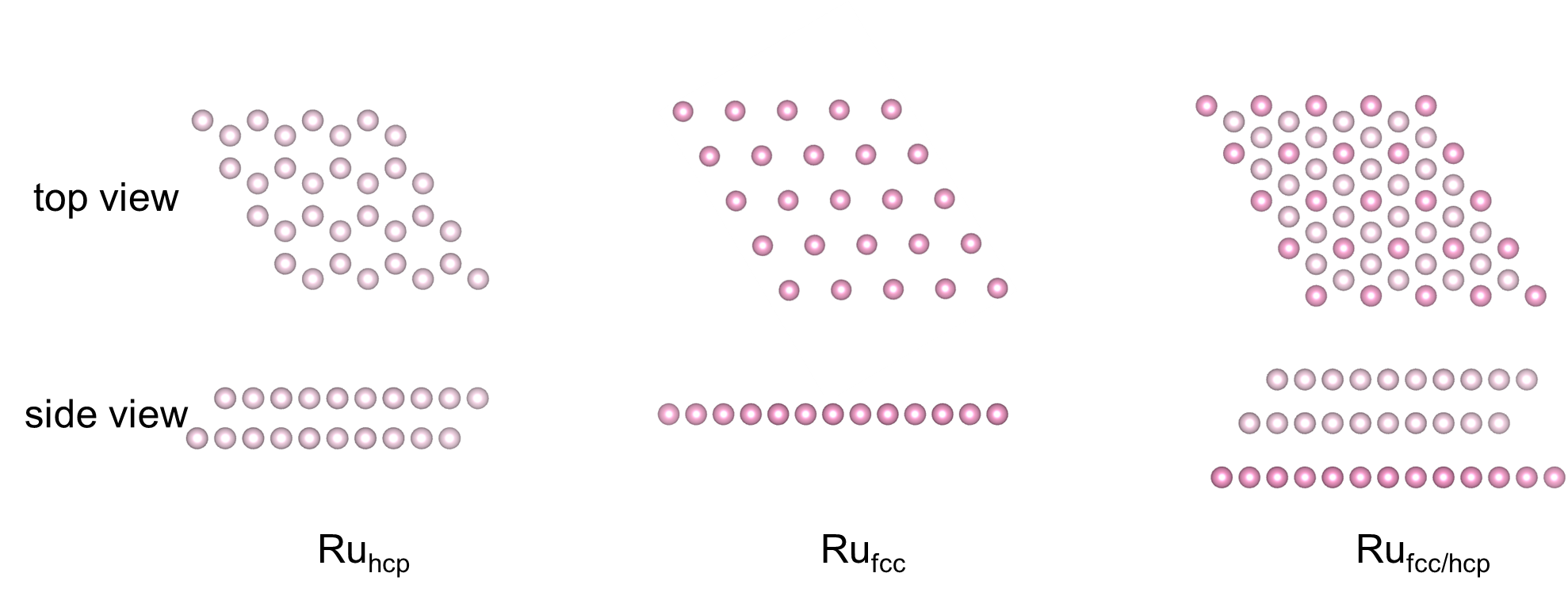


**Figure S26.** Structure models of Ru_hcp_, Ru_fcc_, and Ru_fcc/hcp_. Light red balls represent the hcp phase Ru atoms, while the deep red balls represent fcc phase Ru atoms.


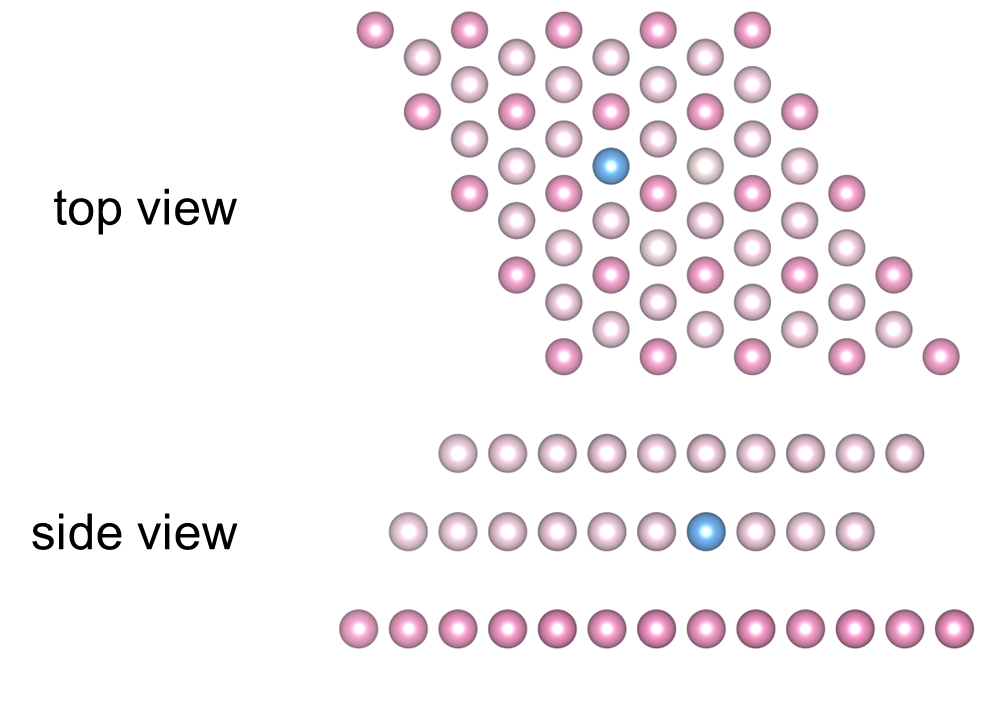


**Figure S27.** Structure model of CrRu_fcc/hcp_. Light red balls represent the hcp phase Ru atoms, while the deep red balls represent fcc phase Ru atoms. Blue ball represents a Cr atom.

**Figure S28.** OH adsorption energy of CrRu_fcc/hcp_ at different active sites.

**Table S1.** The atomic ratios between Cr and Ru in a series of CrRu_fcc/hcp_ aerogels measured by ICP-OES.


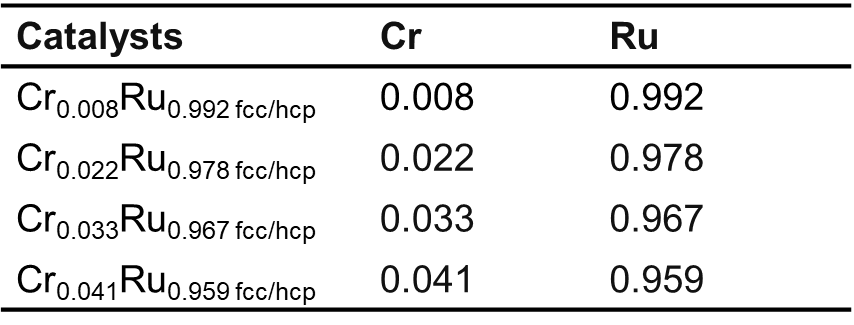


**Table S2**. Comparison of Cr_0.033_Ru_0.967 fcc/hcp_ aerogel with the reported electrocatalysts for HER performance in 1 M KOH.^[5,12,21–26,13–20]^


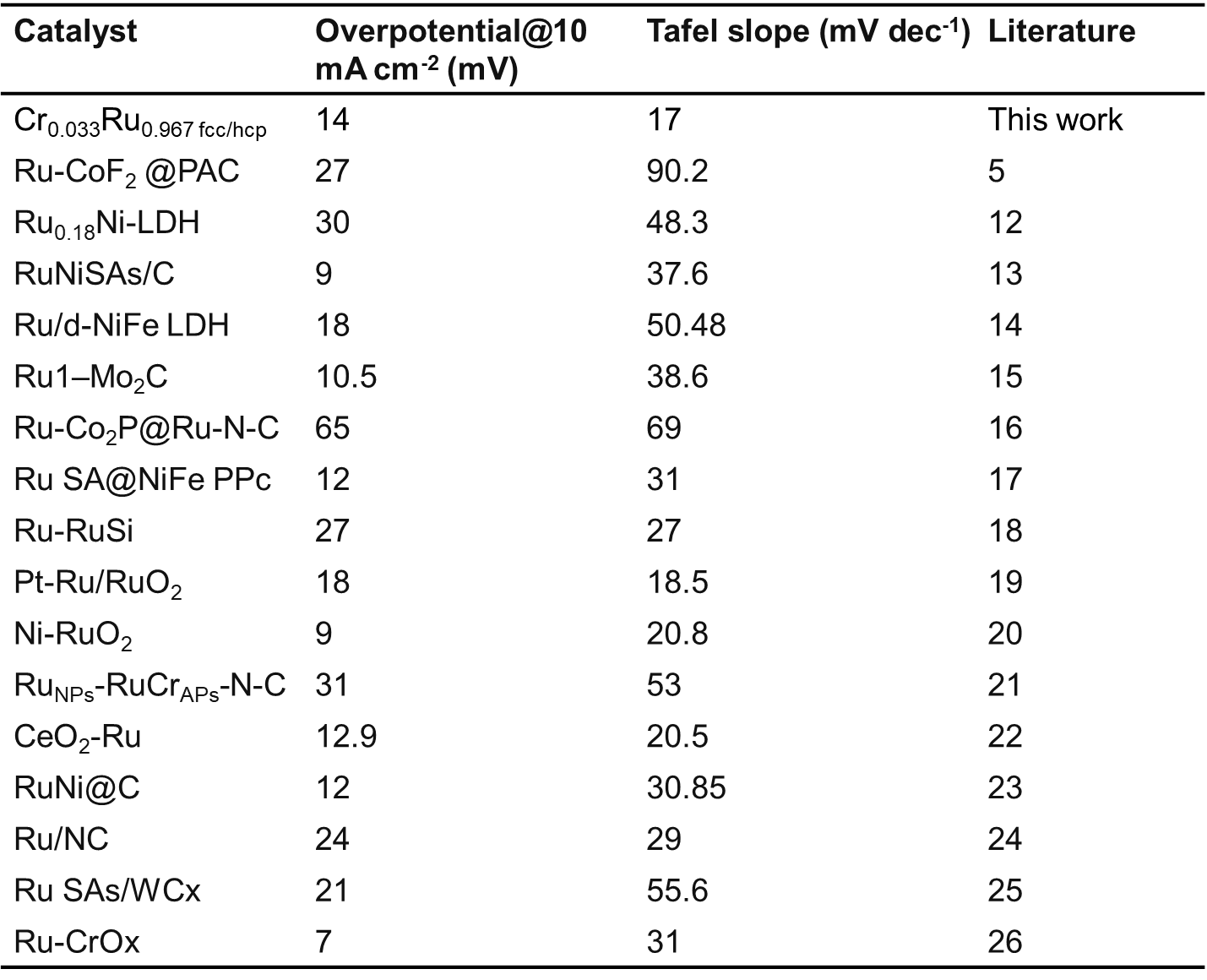


[1] J. Yang, S. Yang, L. An, J. Zhu, J. Xiao, X. Zhao, D. Wang, *ACS Catal.* **2024**, *14*, 3466–3474.

[2] J. Wang, S. Xin, Y. Xiao, Z. Zhang, Z. Li, W. Zhang, C. Li, R. Bao, J. Peng, J. Yi, S. Chou, *Angew. Chemie - Int. Ed.* **2022**, *61*, 1–10.

[3] K. Wang, J. Zhou, M. Sun, F. Lin, B. Huang, F. Lv, L. Zeng, Q. Zhang, L. Gu, M. Luo, S. Guo, *Adv. Mater.* **2023**, *35*, 1–8.

[4] R. Samanta, B. K. Manna, R. Trivedi, B. Chakraborty, S. Barman, *Chem. Sci.* **2023**, *15*, 364–378.

[5] W. Liu, Q. Chen, Y. Shang, F. Liu, R. He, J. Zhang, Q. Li, H. Chai, Y. Tan, S. J. Bao, *Adv. Funct. Mater.* **2024**, *2410325*, 1–9.

[6] D. Joubert, *Phys. Rev. B - Condens. Matter Mater. Phys.* **1999**, *59*, 1758–1775.

[7] I. Nieves-Pírez, A. Muñoz, F. Almeida, V. Blanco, *J. Supercomput.* **2024**, *80*, 16679–16702.

[8] J. P. Perdew, K. Burke, M. Ernzerhof, *Phys. Rev. Lett.* **1996**, *77*, 3865–3868.

[9] A. Allouche, *J. Comput. Chem.* **2012**, *32*, 174–182.

[10] C. R. Hubbard, R. L. Snyder, *Powder Diffr.* **1988**, *3*, 74–77.

[11] X. Zhou, D. Liu, H. Bu, L. Deng, H. Liu, P. Yuan, P. Du, H. Song, *Solid Earth Sci.* **2018**, *3*, 16–29.

[12] N. Shi, R. Ma, L. Lin, W. Xie, P. Liu, P. Li, H. Fan, Y. Tang, Y. Wang, S. Lin, X. Huang, *Small* **2024**, *20*, 1–11.

[13] R. Yao, K. Sun, K. Zhang, Y. Wu, Y. Du, Q. Zhao, G. Liu, C. Chen, Y. Sun, J. Li, *Nat. Commun.* **2024**, *15*, 1–12.

[14] Y. Chen, Y. Liu, W. Zhai, H. Liu, T. Sakthivel, S. Guo, Z. Dai, *Adv. Energy Mater.* **2024**, *14*, 1–10.

[15] T. Chao, W. Xie, Y. Hu, G. Yu, T. Zhao, C. Chen, Z. Zhang, X. Hong, H. Jin, D. Wang, W. Chen, X. Li, P. Hu, Y. Li, *Energy Environ. Sci.* **2024**, *355*, 1397–1406.

[16] P. Wang, K. Wang, Y. Liu, H. Li, Y. Guo, Y. Tian, S. Guo, M. Luo, Y. He, Z. Liu, S. Guo, *Adv. Funct. Mater.* **2024**, *2316709*, 1–12.

[17] Z. Kou, Y. Liu, W. Cui, B. Yang, Z. Li, R. D. Rodriguez, Q. Zhang, C. L. Dong, X. Sang, L. Lei, T. Zhang, Y. Hou, *Energy Environ. Sci.* **2024**, *17*, 1540–1548.

[18] L. Hou, Z. Li, H. Jang, M. G. Kim, J. Cho, W. Zhong, S. Liu, X. Liu, *Angew. Chemie* **2025**, *137*, 1–9.

[19] Y. Zhu, M. Klingenhof, C. Gao, T. Koketsu, G. Weiser, Y. Pi, S. Liu, L. Sui, J. Hou, J. Li, H. Jiang, L. Xu, W. H. Huang, C. W. Pao, M. Yang, Z. Hu, P. Strasser, J. Ma, *Nat. Commun.* **2024**, *15*, 1447.

[20] T. Liu, L. Wang, B. Chen, H. Liu, S. Wang, Y. Feng, J. Zhang, Y. Yin, M. D. Guiver, *Angew. Chemie - Int. Ed.* **2025**, *64*, 1–10.

[21] P. Eskandari, S. Zhou, J. Yuwono, D. Gunawan, R. F. Webster, Z. Ma, H. Xu, R. Amal, X. Lu, *Adv. Mater.* **2025**, *2419360*.

[22] H. Fan, X. Wan, S. Sun, X. Zhou, X. Bu, J. Ye, R. Bai, H. Lou, Y. Chen, J. Gao, J. Zhang, W. Gao, D. Wen, *Adv. Energy Mater.* **2025**, *15*, 1–11.

[23] L. Zhang, H. Hu, C. Sun, D. Xiao, H. T. Wang, Y. Xiao, S. Zhao, K. H. Chen, W. X. Lin, Y. C. Shao, X. Wang, C. W. Pao, L. Han, *Nat. Commun.* **2024**, *15*, 1–12.

[24] Y. Zhu, K. Fan, C. S. Hsu, G. Chen, C. Chen, T. Liu, Z. Lin, S. She, L. Li, H. Zhou, Y. Zhu, H. M. Chen, H. Huang, *Adv. Mater.* **2023**, *35*, 1–8.

[25] X. Lin, W. Hu, J. Xu, X. Liu, W. Jiang, X. Ma, D. He, Z. Wang, W. Li, L. M. Yang, H. Zhou, Y. Wu, *J. Am. Chem. Soc.* **2024**, *146*, 4883–4891.

[26] B. Zhang, J. Wang, G. Liu, C. M. Weiss, D. Liu, Y. Chen, L. Xia, P. Zhou, M. Gao, Y. Liu, J. Chen, Y. Yan, M. Shao, H. Pan, W. Sun, *Nat. Catal.* **2024**, *7*, 441–451.
